# Insulin secretory granules labelled with phogrin-fluorescent proteins show alterations in size, mobility and responsiveness to glucose stimulation in living β-cells

**DOI:** 10.1038/s41598-019-39329-5

**Published:** 2019-02-27

**Authors:** Gianmarco Ferri, Luca Digiacomo, Zeno Lavagnino, Margherita Occhipinti, Marco Bugliani, Valentina Cappello, Giulio Caracciolo, Piero Marchetti, David W. Piston, Francesco Cardarelli

**Affiliations:** 1 NEST - Scuola Normale Superiore, Istituto Nanoscienze - CNR (CNR-NANO), Pisa, Italy; 2 Nanoscopy, Nanophysics, Istituto Italiano di Tecnologia, via Morego 30, 16163 Genoa, Italy; 3 Department of Molecular Medicine, “La Sapienza” University of Rome, Rome, Italy; 4 Department of Cell Biology and Physiology, Washington University School of Medicine, St. Louis, MO 63110 USA; 5 Department of Clinical and Experimental Medicine, Islet Cell Laboratory, University of Pisa, Pisa, Italy; 6 Center for Nanotechnology Innovation@NEST (CNI@NEST), Pisa, Italy

## Abstract

The intracellular life of insulin secretory granules (ISGs) from biogenesis to secretion depends on their structural (e.g. size) and dynamic (e.g. diffusivity, mode of motion) properties. Thus, it would be useful to have rapid and robust measurements of such parameters in living β-cells. To provide such measurements, we have developed a fast spatiotemporal fluctuation spectroscopy. We calculate an imaging-derived Mean Squared Displacement (*i*MSD), which simultaneously provides the size, average diffusivity, and anomalous coefficient of ISGs, without the need to extract individual trajectories. Clustering of structural and dynamic quantities in a multidimensional parametric space defines the ISGs’ properties for different conditions. First, we create a reference using INS-1E cells expressing proinsulin fused to a fluorescent protein (FP) under basal culture conditions and validate our analysis by testing well-established stimuli, such as glucose intake, cytoskeleton disruption, or cholesterol overload. After, we investigate the effect of FP-tagged ISG protein markers on the structural and dynamic properties of the granule. While *i*MSD analysis produces similar results for most of the lumenal markers, the transmembrane marker phogrin-FP shows a clearly altered result. Phogrin overexpression induces a substantial granule enlargement and higher mobility, together with a partial de-polymerization of the actin cytoskeleton, and reduced cell responsiveness to glucose stimulation. Our data suggest a more careful interpretation of many previous ISG-based reports in living β-cells. The presented data pave the way to high-throughput cell-based screening of ISG structure and dynamics under various physiological and pathological conditions.

## Introduction

The lifespan of insulin secretory granules (ISGs) within β-cells, from their biogenesis at the Trans Golgi Network (TGN) to their exocytosis at the plasma membrane, is the result of a complex sequence of molecular signalling events^1^. The dynamic activity of ISGs within β-cells have been suggested to influence the biphasic secretion of insulin^2^, control autophagy^3^, and act as a signalling hub for different membrane proteins during their maturation^4^. Thus, regulation of ISG *structural* (e.g. size, morphology) and *dynamic* (e.g. diffusion, directed motions, and docking) properties is expected to be crucial for their function^5^. In fact, defects in granule structural/dynamic properties are found as hallmarks of pancreatic β-cells dysfunction. For example, it has been suggested that hypercholesterolemia (an hallmark of type-2 diabetes) is associated with an overall increase of granule size accompanied by impaired granule trafficking^6^. Also, very recently, Wan and co-workers demonstrated that older ISGs may change their structural and functional properties through fusion with lysosomes, a process of relevance in type-1 diabetes onset^7^. In spite of the huge interest in such arguments, however, rapid and robust measurement of both structural and dynamic parameters of ISGs in living β-cells has remained a challenging task. On one hand, in fact, current knowledge of ISG structure relies on Transmission Electron Microscopy (TEM), which does not allow dynamic measurements, and can be prone to fixation artifacts^8^. Other structural studies have utilized Structured Illumination Microscopy (SIM), but the relatively slow speed of this approach causes structural information to be convolved with the dynamic properties of ISGs^6^. On the other hand, most of the knowledge about ISG dynamics has relied on Total Internal Reflection Fluorescence (TIRF) imaging and Single Particle Tracking (SPT) analysis. The TIRF approach is limited to the first ~100 nm inside the cell-coverslip interface, revealing ISG trafficking only near the plasma membrane^9–11^. SPT, in principle, extends the spatial scale of the analysis to the whole-cell level and it affords the capability of localizing and tracking multiple objects in a single time-lapse acquisition (for an exhaustive review see ref.^12^). Still, it remains inherently time-consuming and technologically challenging when applied to a three-dimensional (3D) environment where many of the objects are packed closer than the resolution limit of non-super-resolution microscopy, as in the case of labelled ISGs^13–17^.

Spatiotemporal fluorescence fluctuation spectroscopy allows quantitative measurement of average structural and dynamic properties for molecules^18–21^ or sub-cellular organelles^22–24^. This live-cell-imaging approach does not require any preliminary assumptions or knowledge of the system. Information is extracted in the form of a mean square displacement (MSD) versus time-delay plot (hereafter: image-derived MSD, or *i*MSD). A distinguishing feature of *i*MSD is that it yields dynamic information combined with the average size of the moving objects^18,22^. The dynamic properties of the same objects, such as their average diffusivity or mode of motion, are extracted by fitting the *i*MSD trace^22–24^.

Here, we describe the application of the *i*MSD approach to study ISGs in living β-cells. To obtain fluorescent ISGs, we use an EGFP-tagged variant of proinsulin^25^ transiently transfected into INS-1E cells. For each cell, three average parameters of ISGs are extracted from the *i*MSD trace: size, local diffusivity ‘D_micro_’ (D_m_), and anomalous coefficient ‘α’. These values represent a unique point in a 3D parametric space. Clustering of single-cell data points depicts the overall structural and dynamic properties of the selected structure, i.e. the ISG population. We validate the approach by comparing the measured parameters and their alterations with available literature and present a quantitative screening of ISGs size and dynamics under different labeling conditions. To do this, ISGs were labelled with FP fused to peptides or proteins in the granule lumen such as Islet Amyloid Poly-Peptide (IAPP)^26^, Syncollin^27^, and proinsulin, or to a protein embedded in the granule membrane, such as phogrin^28^. Comparing these different labels, we find similar *i*MSD parameters for all the lumenal markers, but highlight a clear shift in the shape and position of phogrin-FP cluster. Of note, phogrin-FP induces a concentration-dependent increase in both granule size and mobility. While the size effect might be expected because of protein overexpression on the granule membrane, the change in mobility appears to be linked to phogrin-dependent depolymerization of the actin meshwork. In addition, phogrin overexpression induces a decrease of cell responsiveness to high-glucose stimulation. These effects have not been previously accounted for, and suggest that previous data on ISG should be re-considered. Given the speed and robustness of *i*MSD, this approach will allow further cell-based screening of ISG structure and dynamics under various physiological and pathological conditions.

## Results

### *i*MSD analysis of the structural and dynamic properties of ISGs in living β-cells

**Figure 1:**
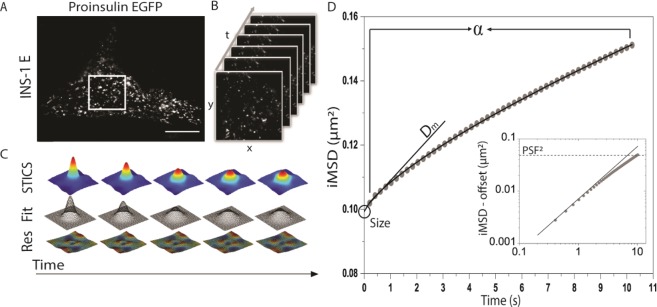
From time-lapse imaging to the iMSD of ISGs in living cells. (**A**) Confocal image of an INS-1E cell transfected with proinsulin-EGFP: the visible punctuated pattern represents the ISGs containing the GFP-tagged C-peptide fragment. (**B**) Acquisition of a cytoplasmic region (white square in A), consisting of 500 frames at 200 ms/frame, 256 × 256 pixels. (**C**) Temporal evolution of the spatiotemporal correlation function obtained by STICS analysis performed on the stack of images in B (top row), with Gaussian fits and residuals (middle and bottom rows). (**D**) iMSD curve obtained by Gaussian fitting of the spatiotemporal correlation functions representing the average diffusion law of the whole population of captured ISGs. The inset contains a log-log plot of the same data. For each experimental curve, values of α, D_m_ and size are extracted by fitting procedures.

**Figure 2:**
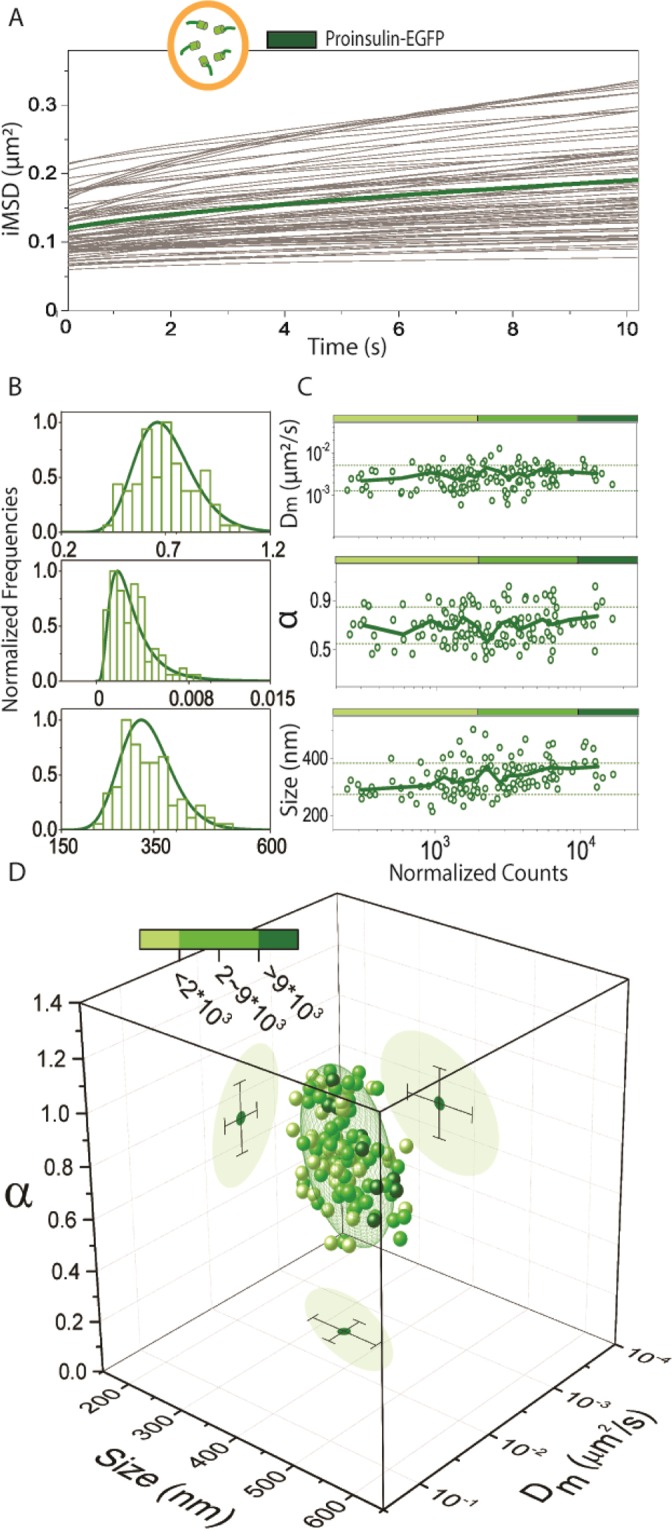
From single *i*MSD traces to whole-cell-population analysis. (**A**) *i*MSD curves from 107 cell acquisitions of proinsulin-EGFP expressing INS-1E cells with the average highlighted in bold green. (**B**) Normalized relative distributions of D_m_, α, and size parameters. (**C**) Protein expression level analysis. Each point in the plots associates, for each acquisition, the obtained value of D_m_ (top), α (middle), and size (low panel) with the protein expression level (expressed in fluorescence counts). Solid green lines represent the average values calculated for each parameter, plus/minus standard deviation (dashed green lines). Solid red lines are calculated by averaging data points with a floating window corresponding to 8 experimental points. Dashed green lines represent standard deviation of all experimental data points. (**D**) Structural and dynamic properties of ISGs labelled with proinsulin-EGFP represented as a scatter plot in which the three values of α, D_m_ and size of each acquisition are organized in a 3-D parametric space. Darkness of the color represents the fluorescence expression level. The 3D-plot projections of data points show the confidence ellipsoids of average values and SD.

The workflow from time-lapse imaging of ISGs to the derivation of their structural/dynamic properties has been carefully assessed and validated in a recently published work^24^ and shown for a typical experiment on ISGs in Fig. 1. We use transient transfection of proinsulin-EGFP into INS-1E cells as a standard procedure to obtain fluorescent, functional ISGs (punctate staining in Fig. 1A). After intra-granular enzymatic processing, the granules contain a dense core of insulin and EGFP-tagged C-peptide^25^. A stack of images is obtained from a 12 × 12 µm region of interest (Fig. 1B) at ~200 ms/frame for at least 100 seconds, while keeping photo-bleaching under control (Suppl. Fig. 1). The spatiotemporal correlation function is calculated by comparing acquired images at increasing time delays (Fig. 1C) following standard spatio-temporal image correlation spectroscopy (STICS) procedures^29^. The width of the peak of the spatial autocorrelation function increases at increasing time delays as a function of the movement of the fluorescent ISGs. By fitting the correlation function, the average *i*MSD of the imaged ISGs is extracted (Fig. 1D). The *i*MSD trace is calculated up to time-lags with maximum length corresponding to 10% of the total acquisition time (~10 seconds in our measurements), as this satisfies statistical criteria for *i*MSD calculation^30^. Extending the time window to larger time-lags increases the spatial scale retrieved (Suppl. Fig. 2, see also ref.^23^). ISG average displacement is linear in time (Fig. 1D inset) only at very short spatiotemporal scales while it substantially deviates from linearity for larger scales^13^. As shown in Fig. 1D, three parameters describe the ISG properties: (*i*) the average diffusion coefficient at short spatiotemporal scales (D_m_; note that this is not the long-range diffusion constant, D_macro_, that depends on the time window analyzed (Suppl. Fig. 2)); (*ii*) the anomalous coefficient also known as ‘α’, which describes the mode of motion of the dynamic object; and (*iii*) the *y*-axis *i*MSD intercept ‘σ_0_^2^’ (subtracted in the *inset* of Fig. 1D), which yields the average apparent size of dynamic objects (i.e. the actual size convolved with the instrumental Point Spread Function, PSF). These three parameters are extracted from *i*MSD traces from 107 cells (Fig. 2A), and analyzed to provide the histogram distributions of whole-cell-populations (Fig. 2B), with the mean ± SD values reported in Table 1. The average structural/dynamic parameters are invariant as a function of the amount of proinsulin-EGFP expressed by INS-1E cells (Fig. 2C). The average apparent ISG size calculated from our experiments is 335 ± 56 nm (Table 1). The analytical procedure for PSF deconvolution (presented in the Supporting Material text) yields an average actual size of ISGs in INS-1E cells of about 220 nm, in good accordance with direct TEM-based quantification of ISG size performed by us (215 ± 39 nm from the analysis of N = 104 single granules from EM micrographs, Suppl. Fig. 3) and by others^8^ on the same cell line. For simplicity, hereafter the apparent sizes directly extracted as *y*-axis *i*MSD intercepts (σ_0_^2^) will be used to compare ISG structural properties across different conditions.

**Table 1:** Summary of *i*MSD-derived parameters.

Cell	Labelling	Size (nm)		D_m_ (μm^2^/s) x10^−3^		α		N		SCD
INS-1E	**Proinsulin-EGFP**	335 ± 56		3 ± 1.7		0.70 ± 0.14		107		/
	**Syncollin-EGFP**	340 ± 60		2.4 ± 1.8		0.71 ± 0.16		48		0.157
	**Phogrin-EGFP**	398 ± 83		8 ± 5		0.55 ± 0.17		72		0.525
	**IAPP-Emerald**	345 ± 73		5 ± 1.5		0.72 ± 0.18		103		0.33
	**Proinsulin-EGFP + Chol**	472 ± 97		11 ± 7		0.39 ± 0.20		71		0.538
	**Proinsulin-EGFP + LatrB**	356 ± 72		10 ± 9		0.46 ± 0.18		65		0.489
	**Phogrin-mCherry + Proinsulin-EGFP**	487 ± 64		14 ± 6.7		0.44 ± 0.12		21		0.91/0.29^*^
	**EGFP-Phogrin**	416 ± 85		16 ± 6		0.62 ± 0.17		58		0.34
	**Proinsulin-EGFP + Glucose**	**Low**	**High**	**Low**	**High**	**Low**	**High**	**Low**	**High**	0.42^**^
		353 ± 64	357 ± 61	2 ± 1	4.6 ± 3.2	0.67 ± 0.12	0.76 ± 0.12	58	54	
	**Mitochondria**	867 ± 101		3.8 ± 2.2		0.64 ± 0.12		70		/
	**Lysosomes**	445 ± 61		8 ± 3.8		0.65 ± 0.12		104		/
	**Clathrin-coated pits**	370 ± 62		13 ± 3.7		0.57 ± 0.11		33		/
MIN6	**Proinsulin-EGFP**	350 ± 69		1.8 ± 1.1		0.78 ± 0.15		66		0.57
βTC3	**Proinsulin-EGFP†**	320 ± 33		1.1 ± 0.7		0.68 ± 0.16		7		/

^*^SCD, statistical cluster distance is calculated between INS-1E phogrin cluster (first value) and INS-1E proinsulin cluster (second value).

^**^SCD, statistical cluster distance is calculated between ISGs labelled by proinsulin-EGFP in INS-1E cells in low- and high-glucose medium.

For validation of our calculated dynamic parameters (α coefficient and D_m_), we can use previous SPT-based studies on granule trafficking. These data typically rely on either confocal-microscopy- or TIRF-based granule imaging and tracking with variable time resolutions, i.e. in the 1–200 Hz range^13–17,31,32^. In particular, recent results suggest that intracellular motions of ISGs follow a subordinated random walk (i.e. sub-diffusion) over the time scale of 1–100 seconds^13^. This is consistent with our results using the *i*MSD both in terms of the average anomalous coefficient (α < 1, indication of sub-diffusive behavior) and of the overall shape of the granule diffusion law. In addition, we probe here average granule isotropic diffusion at a short spatiotemporal scale with local diffusivity D_m_ = (3.2 ± 2.0) × 10^−3^ µm^2^/s, which is in agreement with previous SPT-based data^13–15,33^. The advantage of the *i*MSD algorithm is that it calculates the *average* displacement of all the ISGs in the image, with no need to extract the trajectories of *single* granules, as typically done in a standard SPT experiment (the two methods are compared quantitatively in Suppl. Fig. 4 to show that they yield analogous results if applied to labelled ISGs). The data extracted from *i*MSD analysis can be viewed graphically as a multivariate 3D distribution for σ_0_^2^, D_micro_, and α parameters^23^ for the population of analyzed cells (Fig. 2D). In this 3D parametric space, ISG data are displayed in a characteristic position, which is eventually distinguishable from other sub-cellular structures labelled in INS-1E cells (Suppl. Fig. 5) but, of note, conserved across immortalized β-cell lines (such as MIN-6 and βTC-3 cells, Suppl. Fig. 6). By diagonalizing the covariance matrix of the distribution, we define a confidential ellipsoid that can be regarded as the 3D analog of the confidence interval (mean ± SD) for 1D distributions (Fig. 2A). This allows a global statistical analysis on whole-cell-population data based on the *Mahalanobis* approach^34^, and the statistical cluster distance (Table 1) of each experimental point can be evaluated in comparison to a ‘reference’.

Two experimental conditions were considered to validate the sensitivity of the *i*MSD-based measurements of granule structural/dynamic properties. First, we switched the culture conditions from low (2.8 mM) to high (16.7 mM) glucose concentration. Previous SPT measurements show that granule deployment upon glucose stimulation is the result of two main effects: a 1.5 fold increase in local granule diffusivity combined with an increase in their anomalous coefficient (α of 0.67 in low glucose and 0.76 in high glucose)^14^. Data from our *i*MSD analysis are consistent with these findings (Table 1 and Suppl. Fig. 7). Second validation was made by loading the cells with additional cholesterol. TEM data show that excess cholesterol is delivered specifically to insulin granules causing, on average, a ~1.5-fold granule enlargement^6^. Again, this is mirrored by the results from our *i*MSD analysis: after 1 h treatment with 5 mM cholesterol, ISGs increased ~1.5 folds in size, in average (Table 1 and Suppl. Fig. 7). In our experiments, hypercholesterolemia also induces alteration of the granule dynamic behavior (4-fold increased D_m_, 2-fold decreased α, Table 1 and Suppl. Fig. 7). While this change has not been reported explicitly before, cholesterol-impaired granule trafficking is expected based on the observed retention of syntaxin 6, VAMP4, and clathrin in granule membranes^6^.

### Unveiling the effect of granule labelling on ISG structural and dynamic properties

The commonly used strategy for granule labeling in living cells is genetically-encoded FPs tagged to intrinsic protein markers of the granule. A few different granule-intrinsic proteins are available for this purpose: proinsulin-FP (explained above), the islet amyloid polypeptide (IAPP-FP)^26^, the intralumenal zymogen granule protein, syncollin-FP^27^, and the phosphatidylinositol phosphatase transmembrane protein phogrin-FP^28^. The first three proteins are localized within the granule lumen, while phogrin is a transmembrane protein. Despite their extensive use, little is known about potential overexpression artifacts of these labeling approaches on granule structural/dynamic properties. To elucidate this issue, we used *i*MSD-based analysis of ISGs labeled with each of the four FP constructs in INS-1E cells. The three intraluminal proteins, syncollin-FP, IAPP-FP, and proinsulin-FP, yield similar ISG properties in terms of average size and dynamics of labelled granules (Table 1 and Suppl. Fig. 8).

**Figure 3:**
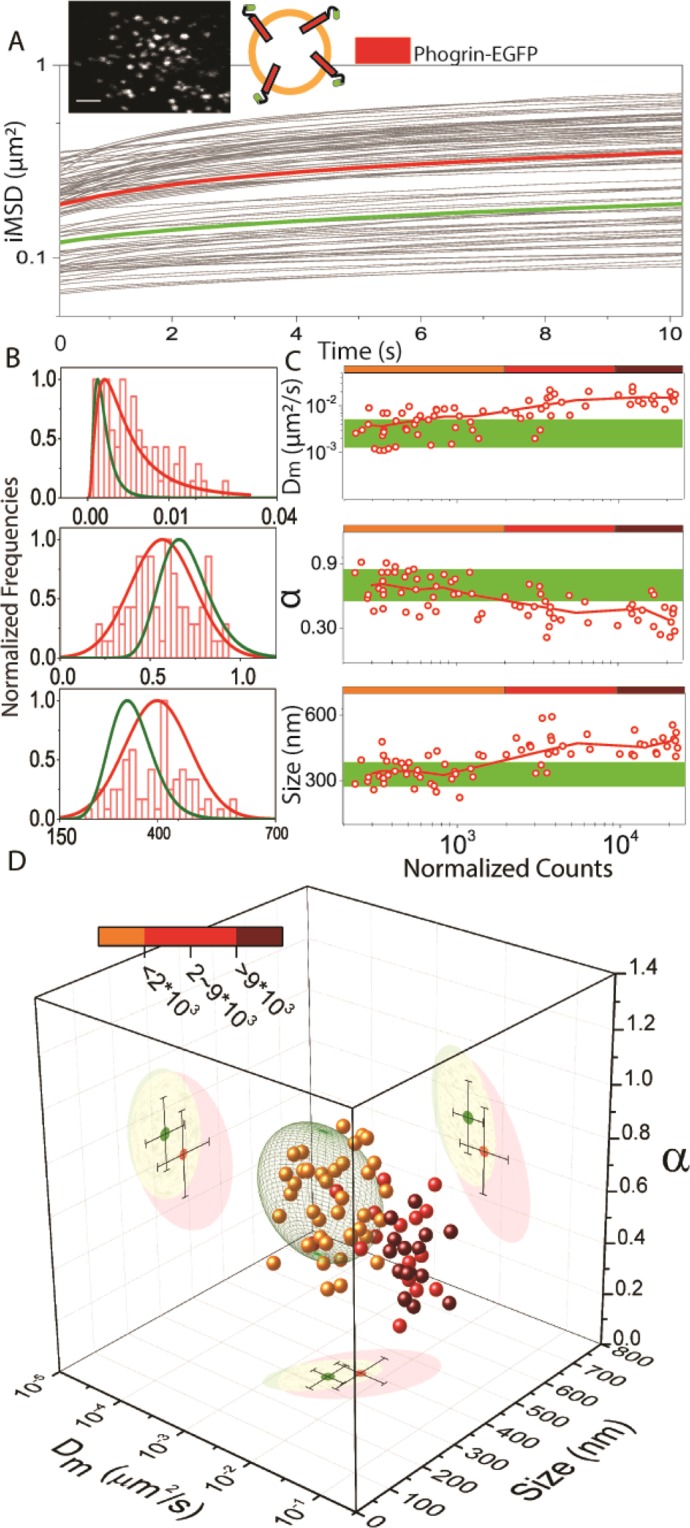
*i*MSD-based analysis of granule alteration induced by labelling. (**A**) On top, example image of a phogrin-EGFP expressing cell (scale bar: 2 µm) and schematic representation of fluorescent protein localization in phogrin-EGFP transfected ISGs. *i*MSD curves of n = 94 acquisitions, with the average highlighted in bold red, as compared to the reference (from Fig. 2) in bold green. (**B**) Normalized relative distributions of α, D_m_ and size for phogrin-EGFP expressing cells. (**C**) Fluorescence expression level analysis. The green area in each plot defines the average position (±SD) of the proinsulin-EGFP reference, as showed in Fig. 2C. Each point associates the obtained value of D_m_ (top), α (middle) and size (low panel) with the protein expression level (expressed in fluorescence counts). Solid red lines are calculated by averaging data points with a floating window corresponding to 8 experimental points. The color-coded bars at the top of each graph indicate the three expression regimes used to classify data in D. (**D**) Structural/dynamic properties of phogrin-EGFP expressing cells as compared to the proinsulin-EGFP reference (represented as a 68% confidence ellipsoid in green). Points are color-coded as a function of the protein expression level defined in C.

**Figure 4:**
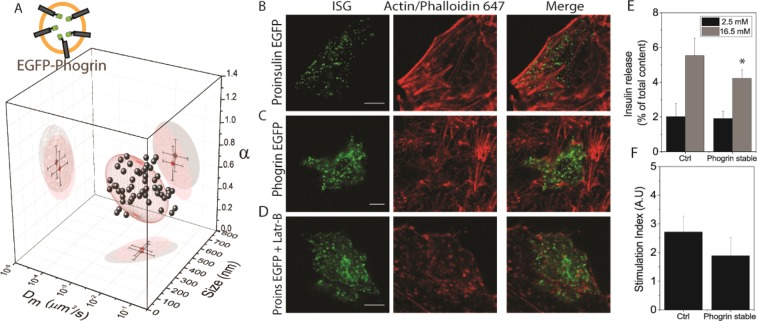
Effect of phogrin overexpression. (**A**) Top left shows a schematic representation of the intragranular localization of the fluorescent protein after transfection of EGFP-phogrin construct (EGFP on the luminal side). Structural/dynamic properties of EGFP-phogrin expressing cells as compared to the phogrin-EGFP (represented as a 68% confidence ellipsoid in red). (**B**) Representative case: proinsulin-EGFP transfected ISGs (green), actin labeled with Phalloidin-647 (red) and overlay of the two channels. (**C**) phogrin-EGFP transfected ISGs (green), actin labeled with Phalloidin-647 (red) and overlay of the two channels. (**D**) Proinsulin-EGFP transfected ISGs (green) and treated with 10 μM Latrunculin-B before fixation, actin labeled with Phalloidin-647 (red) and overlay of the two channels. Scale bar = 5 µm. (**E**) Insulin release (expressed as % of total insulin content) from non-transfected INS-1E cells (Ctrl) and stably expressing phogrin-EGFP INS-1E cells (Phogrin stable) exposed to low glucose concentration (2.8 mM, grey bar) and high glucose concentration (16.7 mM, black bar). *p < 0.05 for Kolmogorov-Smirnov test (n = 6). (**F**) Stimulation index (i.e. ratio between insulin release in high and low glucose condition) for non-transfected INS-1E (Ctrl) and stably expressing phogrin-EGFP (Phogrin stable) INS-1E.

A large majority of the data from these three labels show no dependence on protein concentration, except that, in a few cases, high concentrations of IAPP-FP do elicit a slight increase of average granule size (see Suppl. Fig. 8H,I). This is most likely related to toxic effects on cells, in accordance with what has been reported for COS-1 cells^35^. In contrast to the three intralumenal proteins, transient over-expression of phogrin-EGFP on the ISGs produces a dramatic change in both the structural and dynamic properties of granules, which is readily evident with the *i*MSD analysis (Fig. 3A,B). Cells expressing phogrin-EGFP show ISGs that are, on average, enlarged 1.2 folds as compared to proinsulin-EGFP-labelled ISGs (Table 1). These ISGs also exhibit a ~3-fold higher D_m_ than proinsulin-EGFP-labelled ones (Table 1) and show a strong tendency to be confined by the intracellular environment, with α = 0.55 ± 0.17, as compared to α = 0.70 ± 0.14 of those labelled by proinsulin-EGFP (Table 1). Of note, these differences also depend on the phogrin-EGFP expression level (Fig. 3C). At low phogrin-EGFP expression levels, in fact, the structural and dynamic properties of ISGs are similar to those of the proinsulin-EGFP reference, while alteration becomes clear above a certain concentration threshold for all the three parameters. This is evident in the 3D plot showing color-coded phogrin-EGFP expression levels (Fig. 3D), and average statistical distance between phogrin-FP and proinsulin-FP clusters (Table 1). In Suppl. Fig. 9 we also show that phogrin-FP, in our experimental system, can reach expression levels much higher than its proinsulin-FP counterpart, with similar alteration of the granule structural and dynamic properties. Worthy of note, a substantial increase in granule mobility in the presence of phogrin-FP can be also found in the literature, although it was not recognized as a direct effect of protein overexpression^16^. At this point, to better characterize these observations, we set out to perform a control experiment in which a mCherry-tagged variant of phogrin was co-transfected in INS-1E cells with proinsulin-EGFP and imaged in a two-channel time-lapse experiment (Suppl. Fig. 10). Of note, the *i*MSD analysis of proinsulin-EGFP in these co-transfected cells exhibits enlarged and highly dynamic granules, revealing a dominant effect of the phogrin protein (yellow dots in the 3D plot of Suppl. Fig. 10). The effect of phogrin-FP overexpression on granule size is likely due to the accumulation of transmembrane proteins. This may result in a physical perturbation of the membrane integrity above a critical concentration, similar to the conditions of cholesterol overload on the granule membrane observed here (see Suppl. Fig. 7) or by others^6^. Please note that a similar concentration-dependent alteration of ISGs becomes visible also under stable-transfection conditions (Suppl. Fig. 11). Worthy of mention, present results on granule enlargement are corroborated by analogous observations made by us on a different sub-cellular organelle, the lysosome, under over-expression of GFP-tagged transmembrane protein markers^36^. The effect on granule dynamic properties, on the other hand, prompted us to speculate on a possible effect of phogrin-FP overload on the correct granule docking to the trafficking machinery (e.g. the cytoskeleton). Moving the FP tag from the outer surface to the lumen of the granule yields a similar effect on granule size and mobility (Fig. 4A). We measured actin integrity by using Phalloidin-647 in fixed cells in presence of different granule-specific proteins and experimental conditions (Fig. 4B–D).

In almost all the analyzed cells (N = 30), Phogrin-FP expression induces extensive actin depolymerization (exemplary case in Fig. 4B, additional images in Suppl. Fig. 12A). Notably, this effect is not seen in cells expressing only the proinsulin-EGFP (Fig. 4C, additional images in Suppl. Fig. 12B, N = 25). This phogrin-induced actin phenotype can be artificially reproduced by treating proinsulin-EGFP-expressing cells with an actin-depolymerizing agent, Latrunculin-B (example in Fig. 4D, N = 12)^37^. These results are consistent with the *i*MSD analysis on proinsulin-EGFP expressing cells treated with Lat-B, which shows a ~3-fold increase in granule local diffusivity, a concomitant ~1.5-fold decrease of the α coefficient, and as expected for proinsulin-EGFP, no effect on granule size (Suppl. Fig. 13 and Table 1). In general, to exclude the possibility that Phogrin-FP overexpression is triggering autophagy (and eventually inclusion of granules into autophagosomes) we performed a 2-channel control experiment with autophagosomes labelled by a specific FP-tagged marker (LC3b) and granules labelled by either FP-tagged syncollin and phogrin. Results are included in Suppl. Fig. 14 and demonstrate that autophagy does not significantly contribute to the imaging of ISGs in our experimental system.

As a final test, we evaluated insulin secretion from the phogrin-FP expressing stable cells and compared it to unlabeled cells. In case of low-glucose concentrations (2.8 mM), insulin release appears to be unaffected by phogrin-FP expression (Fig. 4E). In contrast, high-glucose concentrations (16.7 mM) showed a 30% reduction in normalized insulin secretion from phogrin-FP expressing cells as compared to unlabeled cells (Fig. 4F).

## Discussion

The proper intracellular function of ISGs depends on both structural (e.g. size) and dynamic (e.g. diffusion mode) properties (1). Defects in granule structural/dynamic properties are recognized hallmarks of pancreatic β-cells dysfunction such as those found under hypercholesterolemia conditions^6^. Despite the importance of these ISG properties, no currently utilized method provides simultaneous, rapid, and robust access to both structural and dynamic information of ISGs in living cells. Electron microscopy achieves high spatial resolution at the expenses of dynamic information (and can be prone to fixation artifacts)^8^. On the other hand, approaches such as SPT provide valuable dynamic information but typically underexploit the structural one. These methods are also limited by the time-consuming extraction of trajectories and challenges inherent with a crowded 3D environment where many objects are packed within the microscopy resolution limit^13,15,17^.

This work exploits the simultaneous estimation of the structural (size) and dynamic (diffusivity and mode of motion) properties of ISGs by *i*MSD-based spatiotemporal correlation spectroscopy in living β-cells. The *i*MSD approach, originally introduced to study molecules^18,21^, and recently adapted to study endocytic vesicles^23^, is applied here to insulin secretory granules. The method achieves high temporal resolution at the expense of distinguishing local heterogeneities, yielding quantitative information at the whole-cell level (without the need to extract individual trajectories). This becomes particularly evident if results reported here are compared to SPT-based analyses from the literature. It is interesting to note, for instance, that Hoboth and co-workers report on the co-existence in the cell of three granule sub-populations, defined as “highly dynamic” (D∼10^−2^ µm^2^/s), “restricted” (D∼10^−3^ µm^2^/s), and “nearly immobile” (D ∼10^−4^ µm^2^/s), with the latter two populations being far more abundant^15^. As expected, here, averaging the whole population of granules by *i*MSD, we obtain an overall diffusivity that is in the order of 10^−3^ µm^2^/s. It shall be kept in mind that, as any other fluctuation-based statistical method, also *i*MSD could be in principle biased towards the detection of larger/brighter objects. We excluded such possibility for experiments on labelled ISGs by cross-correlating *i*MSD results on size and dynamics with those obtained, independently, by TEM, optical microscopy and SPT: nicely, it is concluded that no clear correlation is present between the size/intensity of granules and their diffusivity (data reported in Suppl. Figs 3 and 4).

Exploiting the high-throughput potential of the *i*MSD method, we measure structural and dynamic features of whole cell populations of trafficking granules, in particular under different labelling conditions (i.e. protein overexpression). We find similar *i*MSD-derived clusters for all lumenal markers, but a clear shift in the shape and position of the phogrin-FP cluster. In our experiments, phogrin-FP induces a concentration-dependent increase in both granule size and mobility. The size effect is consistent with steric hindrance due to protein overexpression on the membrane. However, the change in mobility appears to be linked to phogrin-dependent depolymerization of the membrane actin meshwork. It has been proposed that phogrin functions to reduce plasma membrane phosphatidylinositol 4,5-bisphosphate (PI(4,5)P2) abundance, which, in turn, induces actin depolymerization^28,38^. In addition, we report that phogrin overexpression induces a substantial decrease (~30%) of insulin secretion under high glucose stimulation. Our measured biophysical size and dynamics parameters using phogrin-FP labeled cells are consistent with the secretion data, which give added confidence in our results. Still, it is not clear how to reconcile existing contradictory literature in which different cell lines and experimental conditions have been tested, and phogrin overexpression was alternatively associated with suppression of glucose-stimulated insulin secretion, its stimulation, or even with no substantial effect^39^. The decreased insulin secretion from stably-transfected cells reported here suggests that the impact of phogrin overexpression is limited to cell responsiveness at high glucose concentrations. We does not see phogrin dependent variations at lower concentrations, and that my also explain some of the disparate data in the literature.

Granule-specific labelling effects have not been previously described as an explicit parameter in β-cell function. Our data suggest that it will be useful to re-interpret and evaluate many previously published results. We expect that the information obtained with *i*MSD analysis can be increased by combining it with ‘intelligent’ dyes/proteins to simultaneously probe selected parameters on the structure of interest (e.g. pH, membrane order, etc.). Such implementation could transform the basic strategy presented here in a flexible, multiplexed platform to quantitatively address the complex regulation of insulin granule trafficking.

## Materials and Methods

### Cell culture, transfection and fluorescence staining

INS-1 E cells^40^ were maintained in culture at 37 °C, 5% CO_2_ in RPMI 1640 medium containing 11.1 mM D-glucose, 10 mM Hepes, 10 mM L-Glutamine, 100 U/ml penicillin-streptomycin, 10 mM sodium-pyruvate, 50 µM tissue culture grade β-mercaptoethanol (all purchased from Life Technologies, Thermo Fisher). Before live-cell imaging, cells were plated onto IbiTreat µ-Dish 35 mm, high walls, #1.5 polymer coverslip, tissue culture treated, sterilized and fluorescence microscopy suitable (Ibidi, Martinsried, Germany). INS-1E cells were transfected using lipofectamine 2000 reagent as per manufacturer’s instructions and using Optimem culture medium (Life Technologies, Thermo Fisher). Mitochondria and Lysosomes staining in INS-1E cells were performed using MitoTracker-Green FM and Lysotracker Red dnd-99 (Thermo Fisher), following the manufacturer’s instructions. Transferrin-Alexa Fluor® 488 (Thermo Fisher) was used to label clathrin-mediated endocytosis. INS1- E cells were incubated in medium containing 20 nM of transferrin conjugate at 37 °C for 20 minutes before imaging. To generate a stably Phogrin-EGFP expression line of INS-1E, cells were transfected with Lipofectamine 2000, as previous described here, with Phogrin-EGFP plasmid, carrying NeoR gene which confers resistance to Geneticin (G418). 48 h after transfection, the medium was replaced with fresh complete medium supplemented with G418 at 50 μg/mL and a 15 days selection allowed obtaining a stable polyclonal cell population expressing Phogrin-EGFP localized in ISGs. After selection, G418 concentration was reduced at 10 μg/mL as maintenance for 1 week. The day before experiment, 5 × 10^5^ stably transfected cells were seeded onto IbiTreat µ-Dish in complete medium devoid of G148.

Insulinoma cell line MIN6^41^ were cultured at 37 C and 5% CO_2_ in Dulbecco’s modified eagle medium (DMEM) supplemented with 25 mM glucose, 10% fetal bovine serum (FBS) and penicillin/streptomycin. Cells were grown in these conditions on 29 mm Glass bottom dish with 20 mm micro-well (#D29-20-1.5-N, Cellvis) for 24 hrs or to 40–50% confluency prior to transfection with Effectene transfection reagent (Qiagen). After a 24-hrs of incubation, the cells were ready to be imaged. βTC3 cells were a kind gift from Cris Brown in Fumihiko Urano’s lab at Washington University in St. Louis. They were cultured in high glucose RPMI 1640 medium supplemented with 2 mM L-glutamine, 10% fetal bovine serum, 100 μg/ml penicillin, and 50 μg/ml streptomycin at 37 °C under an atmosphere of 5% CO_2_. Cells were grown in these conditions on 29 mm Glass bottom dish with 20 mm micro-well (#D29-20-1.5-N, Cellvis) for 24 hrs or to 40–50% confluency prior to transfection with Effectene transfection reagent (Qiagen). After 24 hrs of incubation, the cells were ready to be imaged.

### Plasmids

Proinsulin-EGFP was cloned as described in ref.^42^. Phogrin-EGFP plasmid was obtained subcloning EGFP coding sequence from EGFP-N3 plasmid (Clonetech) by PCR and introducing AgeI (ACCGGT) and BsrGI (TGTACA) restriction enzyme sites at 5′ and 3′ ends, respectively. (Forward primer: 5′-gactggACCGGTcgccaccatggtgagcaagggcgagg-3′; Reverse primer: 5′-cctgggTGTACAgctcgtccatgccgagag-3′). These sites were used to clone the fragment in phogrin-mCherry plasmid^43^ by double-digestion with the same enzymes to remove mCherry sequence and subsequent ligation. Syncollin-EGFP plasmid was a kind gift of Michael Edwardson (Department of Pharmacology, University of Cambridge). IAPP-Emerald plasmid was a kind gift of Erik Renström (Department of Clinical Sciences, Lunds University). EGFP-Phogrin plasmid was cloned starting from Phogrin cDNA sequence, EGFP was inserted three amino acid before the KK cleavage site by PCR.

### Actin staining

For actin staining experiments, cells were fixed with 4% paraformaldehyde (PFA) 24 h after transfection and stained for actin cytoskeleton with Phalloidin-647 (Life Technologies, Thermo Fisher) following standard protocol. In case of Latrunculin-B treatment, cells were incubated with 10 μM Latrunculin-B for 40 minutes prior to fixation.

### Drug treatments

For cholesterol overloading, cells were incubated with 5 mM CHOL (soluble cholesterol from Sigma; 1 g cholesterol-MβCD complex contains approximately 40 mg cholesterol) at 37 °C for 1 h. In order to disrupt the actin meshwork at the plasma membrane, transfected cells were treated with 10 μM Latrunculin-B (Sigma-Aldrich) for 15 minutes before live cell imaging. For glucose-stimulation experiment, standard RPMI culture media was replaced with RPMI media containing 2.8 mM glucose after 24 hours and grown for a further 24 hours. Cells were equilibrated in a low-glucose buffered solution (HBSS: 114 mM NaCl, 4.7 mM KCl, 1.2 mM KH_2_PO_4_, 1.16 mM MgSO_4_, 20 mM Hepes, 2.5 mM CaCl_2_, 25 mM NaHCO_3_, 2.8 mM glucose, 2% BSA, pH 7.2) for 2 hours at 37 °C. Subsequently, RPMI complete medium supplemented with 16.7 mM glucose was added to dishes before transferring the plates to the microscope for live-cell imaging.

### Insulin secretion assay

Insulin secretion was measured as previously described^44^. Briefly, INS-1E cells were exposed to glucose-free RPMI medium for 2 h and then, after a 30 min washing period with a Krebs’ solution containing 2.8 mM glucose, they were exposed to 2.8 or 16.7 mM glucose for 30 min. At the end, the supernatant was collected and stored at −20 °C. Total insulin content was extracted with an acid ethanol solution and insulin levels were quantified by the High Range Rat Insulin ELISA kit following the manufacturer’s protocol (Mercodia AB, Uppsala, Sweden).

### Fluorescence microscopy

Fluorescence microscopy experiments on INS-1E transfected cells, bathed in 11 mM glucose RPMI-1640 complete medium, were carried out with a Leica SP5 inverted confocal microscope. Images were acquired illuminating the sample with a 488-nm laser for EGFP and Emerald excitation and 561-nm laser for mCherry excitation, using a 40× (N.A. 1.27) oil-immersion objective. EGFP fluorescence was collected between 500 and 600 nm, while mCherry fluorescence was collected between 570 and 670 nm. Each acquisition consists in a collection of 500 frames (256 × 256 pixels) at a sampling frequency of 1400 Hz/lines (~3 µs/pixel) and with a pixel size of 50 nm. Pinhole aperture was set to 1 Airy. Experiments on MIN6 cells were performed with an inverted Zeiss LSM 880 confocal microscope. The objective used is a Zeiss Fluar 40×/1.30 NA Oil M27, the acquisition time per each 256 × 256-pixels frame was 202.5 ms. The total field of view was 12.5 microns. Cells were kept at 37 C and 5% CO_2_ thanks to a PECON stage-top incubator. Experiments were performed acquiring 500 frames with no signal averaging, for a total time of a minute and 41 seconds. Photobleaching experiments were carried out before the acquisition of individual plates to determine the optimal excitation intensity on the sample. Both phenol-free DMEM and KRBH buffer with 13 mM glucose concentration were used as imaging buffers, but no significant differences were noticed after the analysis of the data. Imaging experiments on actin cytoskeleton stained with Phalloidin-647 were performed on Zeiss LSM 800, using a 63×/1.47 Oil objective and collecting fluorescence emission of transfected ISGs (EGFP) on 500–600 nm interval and on 650–750 nm interval for Phalloidin-647 emission. In all experiments, a low laser illumination power at the objective was used (typically 0.5–1 µW) to keep phototoxicity under control.

### Image processing and data analysis

Main theoretical framework of *i*MSD analysis can be found in^18^ for molecules diffusing on cell membranes, and in^22–24^ for studying the motion of nanostructures in cell cytoplasm. Briefly, each time-lapse acquisition (N = 500 frames) was analyzed using a custom script working on MATLAB (MathWorks Inc., Natick, Ma) (for more details on the script see^24^) which computes by Fast Fourier methods the spatiotemporal correlation function, defined as follows:

1 $$g(\xi ,\eta ,\tau )=\frac{\langle i(x,y,t)i(x+\xi ,y+\eta ,t,+t)\rangle }{{\langle i(x,y,t)\rangle }^{2}}-1$$

*g*(*ξ*, *η*, *τ*) can be fitted with a standard Gaussian function of the form:

2 $$g(\xi ,\eta ,\tau )={g}_{0}+{g}_{1}(\tau )\exp \{-\frac{{({\rm{\xi }}-{{\rm{v}}}_{{\rm{\xi }}}{\rm{\tau }})}^{2}+{({\rm{\eta }}-{{\rm{v}}}_{{\rm{\eta }}}{\rm{\tau }})}^{2}}{{{\rm{\sigma }}}^{2}({\rm{\tau }})}\}$$

whose variance σ^2^(τ) is analogous to the mean square displacement extracted directly from imaging, *i*MSD. Each *i*MSD curve was fitted with the following equation to define the diffusion law and to extract the diffusion parameters α, D_micro_, and σ^2^. Please note, as already reported in a previous work^45^, that in all the experiments on labelled granules the peak of the spatiotemporal correlation function remains centered at (0, 0) while increasing in width. This reflects the diffusive behavior of granules, while excluding net collective movements, such as those eventually produced by whole-sample drifts.

3 $${\sigma }_{r}^{2}(\tau )=\kappa {\tau }^{\alpha }+{\sigma }_{0}^{2}$$

4 $${\sigma }_{r}^{2}(\tau )\cong \frac{{L}^{2}}{3}(1-\exp (-\frac{\tau }{{\tau }_{c}}))+4{D}_{macro}\tau +{\sigma }_{0}^{2}$$

With Eq. (3) the ISG’s motion is categorized according to the value of *α* in (*α* = 1) isotropic diffusion, (*α* < 1) anomalous diffusion, (*α* > 1) guided diffusion. In case of (partially) confined diffusion (4) is used to fit σ^2^(τ) trend, where L defines the linear size of the confinement area, *τ*_*c*_ is an index of how fast confinement occurs, *D*_*macro*_ is the diffusivity at large time scale and represents ¼ of the derivative of σ^2^ for *τ* → ∞. *D*_*micro*_ is calculated by the slope of σ^2^ for *τ* → 0, it represents the diffusivity inside the confinement area and is defined by the following relation:

5 $${D}_{micro}={D}_{macro}+\frac{{L}^{2}}{12{\tau }_{c}}$$

In both [3] and [4], ${\sigma }_{0}^{2}$ is the intercept value which is related to the average particle size, as already discussed in [2]. In particular, the apparent particle size could be calculated using:

6 $$Siz{e}_{app}=\sqrt{{\sigma }_{0}^{2}}$$

In this case, *Size*_*app*_ (apparent) represents the average diameter of imaged ISGs, *i.e*. the real size of the ISGs convolved with instrument’s PSF. For the derivation of the actual size, refer to equations presented in Supplementary Material. The PSF at 488 nm was calibrated using 30-nm fluorescent beads and resulted to be 270 nm.

### Cluster similarity analysis

The measured parameters (i.e. the short-scale diffusion coefficient D, the *i*MSD intercept value σ^2^_0_ and the anomalous coefficient α) of each image-stack define a data point in a 3-dimensional space. Thus, the set of data points corresponding to the dynamics of a specific system is a 3D multivariate distribution of the measured values. To quantify a degree of similarity among the investigated dynamics, we calculated the statistical difference d between two distributions, as follows:

7 $$d=\sqrt{C{({\mu }_{1}-{\mu }_{2})}^{T}{{\rm{\Sigma }}}^{-1}({\mu }_{1}-{\mu }_{2})}$$

where C is a scale factor, *μ*_1_ and *μ*_2_ are three-component vectors representing the mean values of the first and second distribution, respectively. ∑ is defined in terms of the corresponding covariance matrices, ∑_1_ and ∑_2_:

8 $${\rm{\Sigma }}=\frac{{{\rm{\Sigma }}}_{1}+{{\rm{\Sigma }}}_{2}}{2}$$

Equation (1) generalizes the Mahalanobis distance between a point and a distribution and represents a measurement of statistical distance that take into accounts extents, relative positions and orientations of the observed distributions in the parameter-space. For a single distribution, a confidence volume can be computed from the covariance matrix and is represented as an ellipsoid. The ellipsoid is therefore defined by the distribution itself; its location, size and orientation, depend on averages and standard deviations of the observed variables. The scale factor in Eq. 1 is related to the dimensionality of the problem and can be normalized (i.e. C = 1.1396) to ensure that the statistical meaning of the ellipsoid represent the 3D generalization of the error bars, which are usually employed for 1D distribution. In other words, 68% of the observed data falls within the ellipsoid. In terms of statistical distance, with this normalization, two distributions with the same mean values have d = 0, independently of their covariance matrices. If the average values are different and the corresponding ellipsoids intersect each other, then 0 < d < 1. If the ellipsoids are externally tangent, then d = 1. If the distributions are far enough that there is no intersection between the ellipsoids, then d > 1. Finally, an overlap coefficient between two distributions can be estimated. We employed the definition of the Szymkiewicz-Simpson coefficient^46^ and calculated the overlap as follows:

9 $$s=\frac{{{\rm{\Omega }}}_{0}}{{\rm{\min }}\,\{{{\rm{\Omega }}}_{1},{{\rm{\Omega }}}_{2}\}}$$

where Ω_1_ and Ω_2_ are the volumes of the two ellipsoids and Ω_0_ represents the volume of their intersection.

### Fluorescence-based expression level analysis

Average fluorescence intensity was calculated on the first frame of each acquired movie, by means of ImageJ plugin Analyze Particles, used to isolate ISGs fluorescence spots. The obtained value was normalized based on the laser intensity and PMT gain of each acquisition using green Autofluorescent Plastic Slides from CHROMA® as a reference. For IAPP-Emerald fluorescence intensity analysis, fluorescence counts are firstly normalized to EGFP counts using brightness ratio between the two FP.

### Trajectory analysis

Trajectories analysis was performed using TrackMate plugin for ImageJ. LogDetector algorithm was used to detect fluorescence spots; Lap Tracker algorithm was used to perform the analysis of trajectories. MSDs were computed using a custom made Matlab tool.

### Granule morphometric analysis from TEM micrographs

INS-1E cells were grown on Petri dishes till confluence. For the ultrastructural analysis of insulin granules produced by cells we developed a conventional embedding procedure as previously described^47^. Briefly INS-1E cells monolayers were fixed in a 1.5% glutaraldehyde solution in Na cacodylate buffer 0.1 M pH 7.4, for 1 h at room temperature. Next, cells were scraped, collected and centrifuged at 13200 rpm and RT for 15 min in the same fixative solution, until a visible pellet was obtained, and kept in new fixative solution overnight at 4 °C. Then samples were post-fixed with reduced osmium tetroxide (1% OsO4 plus 1% K3Fe(CN)6 in the same buffer, rinsed, stained en bloc with 3% solution of uranyl acetate in 20% ethanol, dehydrated and embedded in epoxy resin (Epon 812, Electron Microscopy Science, Hatfield, PA, USA) that was then baked for 48 h at 60 °C. For the TEM analysis, thin 90-nm sections were cut using a UC7 (Leica Microsystems, Vienna, Austria). Sections were examined with a Zeiss LIBRA 120 plus transmission electron microscope equipped with an in-column omega filter. Electron digital micrographs (2048 × 2048 16 bit) were used for the evaluation insulin granules diameter using the software Fiji.

## Supplementary information

Supplementary Information and Figures
