# Supplementary material for: Insulin secretory granules labelled with phogrin-fluorescent proteins show alterations in size, mobility and responsiveness to glucose stimulation in living β-cells
Source: Sci Rep. 2019 Feb 27;9:2890. doi: 10.1038/s41598-019-39329-5 (PMC6393586; doi:10.1038/s41598-019-39329-5)
Supplement: Supplementary file 1 — Supplementary Information and Figures [file 41598_2019_39329_MOESM1_ESM.pdf]

Title:

Insulin secretory granules labelled with phogrin-fluorescent proteins show alterations in size, mobility and responsiveness to glucose stimulation in living β-cells

Authors:

Gianmarco Ferri [a,b], Luca Digiacomo [c], Zeno Lavagnino [d], Margherita Occhipinti [e], Marco Bugliani [e], Valentina Cappello [f], Giulio Caracciolo [c], Piero Marchetti [e], David W. Piston [d], and Francesco Cardarelli [a,*]

Affiliations:

[a] NEST - Scuola Normale Superiore, Istituto Nanoscienze - CNR (CNR-NANO), Pisa, Italy.

[b] Nanoscopy, Nanophysics, Istituto Italiano di Tecnologia, via Morego 30, 16163 Genoa, Italy

[c] Department of Molecular Medicine, "La Sapienza" University of Rome, Italy.

[d] Department of Cell Biology and Physiology, Washington University School of Medicine, St. Louis, MO, 63110, USA

[e] Department of Clinical and Experimental Medicine, Islet Cell Laboratory, University of Pisa, Pisa, Italy.

[f] Center for Nanotechnology Innovation@NEST (CNI@NEST), Pisa, Italy

[*] To whom correspondence should be addressed: francesco.cardarelli@sns.it

Keywords:

fluorescence correlation spectroscopy, *i*MSD, Insulin Secretory Granule, INS-1 E, human β-cells, EGFP, Proinsulin, Phogrin, IAPP, Syncollin, MIN6, βTC3

Supporting Information and Figures

***i*MSD curve: intercept value and particle size**

Instrumental waist

A Gaussian point spread function (PSF) is defined as [(1), Eq. 2.6]:

$$W(\xi, \eta, \zeta) = \frac{1}{w^2 w_z^2 (2\pi)^{3/2}} exp\left\{-2\left(\frac{\xi^2+\eta^2}{w^2} + \frac{\zeta^2}{w_z^2}\right)\right\} \quad (1)$$

Where w and $w_z$represent the waist of the PSF along radial and axial direction, respectively. Fig. 1 shows (left panel) the section of the PSF over the plane $\zeta = 0$ and (right panel) the profile

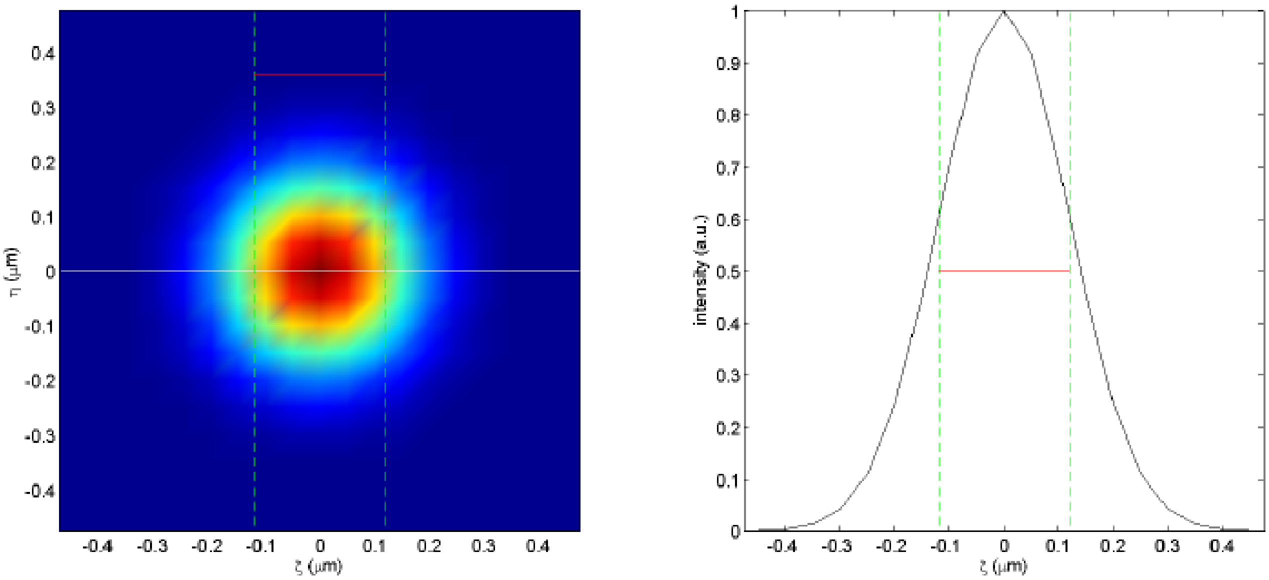

Figure 1: (left) Representative PSF on the plane $\zeta = 0$ and (right) profile of the PSF along a radial direction. The red segment indicates the radial waist w (here $w = 238$ nm).

$W(\xi, 0,0)$. The radial waist w is indicated as a red segment and can be regarded as the "size" of the PSF on the focal plane.

Intercept of the *i*MSD curve

$g(\xi, \eta, \zeta, \tau)$ is defined as the spatiotemporal image correlation, as function of the spatial lag-variable ξ, η, ζ and the temporal lag-variable τ. We can define the position vector $\vec{\rho} \equiv (\xi, \eta, \zeta)$ and – for point-like particles- we can express $g(\vec{\rho}, \tau)$ as [(1), Eq. 2.5]:

$$g(\vec{\rho},\tau) \propto P(\vec{\rho},\tau) \otimes [W(\vec{\rho}) \otimes W(\vec{\rho})] \quad (2)$$

Where $P(\vec{\rho},\tau)$ is the probability density function of particle displacement, it depends on the investigated dynamics and determines the time evolution of g. $W(\vec{\rho})$ is the PSF and its Gaussian approximation is given in Eq. 1.

For 2D motion, g can be approximated as a Gaussian surface of variance $\sigma^2(\tau)$ [(1), Eq. 2.9]. Under this assumption, $P(\vec{\rho},\tau)$ regulates the trend of $\sigma^2(\tau)$, whose boundary value $\sigma^2(0)$ is fixed by $W(\vec{\rho})$ through the radial waist of the PSF. In other words,

$$g(\vec{\rho},0) \propto \left\{-\frac{\rho^2}{\sigma^2(0)}\right\} = exp\left\{-\frac{\rho^2}{w^2}\right\} \propto W(\vec{\rho}) \otimes W(\vec{\rho}) \quad (3)$$

Eq. 3 can be evaluated by combining Eq. 2 and Eq. 1 and by considering that $P(\vec{\rho},0) = \delta(\vec{\rho})$, where δ is the Dirac delta function. Hence, the intercept of the *i*MSD curve is $\sigma^2(0) = w^2$.

Contribution of particle size.

When the particle under study has more than one fluorophore and its size is not negligible, $g(\xi,\eta,\tau)$ must include also the spatial extension of the particle itself. If we define $S(\vec{\rho})$ as the spatial distribution of the fluorophores on the particle surface,

$$g(\vec{\rho},\tau) \propto P(\vec{\rho},\tau) \otimes [W_1(\vec{\rho}) \otimes W_1(\vec{\rho})] \quad (4)$$

Where

$$W_1(\vec{\rho}) = W(\vec{\rho}) \otimes S(\vec{\rho}) \quad (5)$$

And W is the PSF. For 2D Motion, we can focus on a radial profile, then generalize the solution over the (ξ,η)-plane. In this regard, by assuming a uniform distribution of the fluorophores, $S(\xi,0)$ can be written as:

$$S(\xi,0) = \begin{cases} \kappa & for\ |\xi| < R \\ 0 & otherwise \end{cases} \quad (6)$$

Where R is the particle radius and κ is a constant normalization factor. By combining Eq. 1, 5 and 6, we obtain:

$$W_1(\xi,0) = W(\xi,0) \otimes S(\xi,0) = \kappa \int_{-R}^{R} exp\left\{-2\frac{(\xi+\xi')^2}{w^2}\right\} d\xi' \propto \left[erf\left\{\frac{\sqrt{2}(\xi+R)}{w}\right\} - erf\left\{\frac{\sqrt{2}(\xi+R)}{w}\right\}\right]$$

(7)

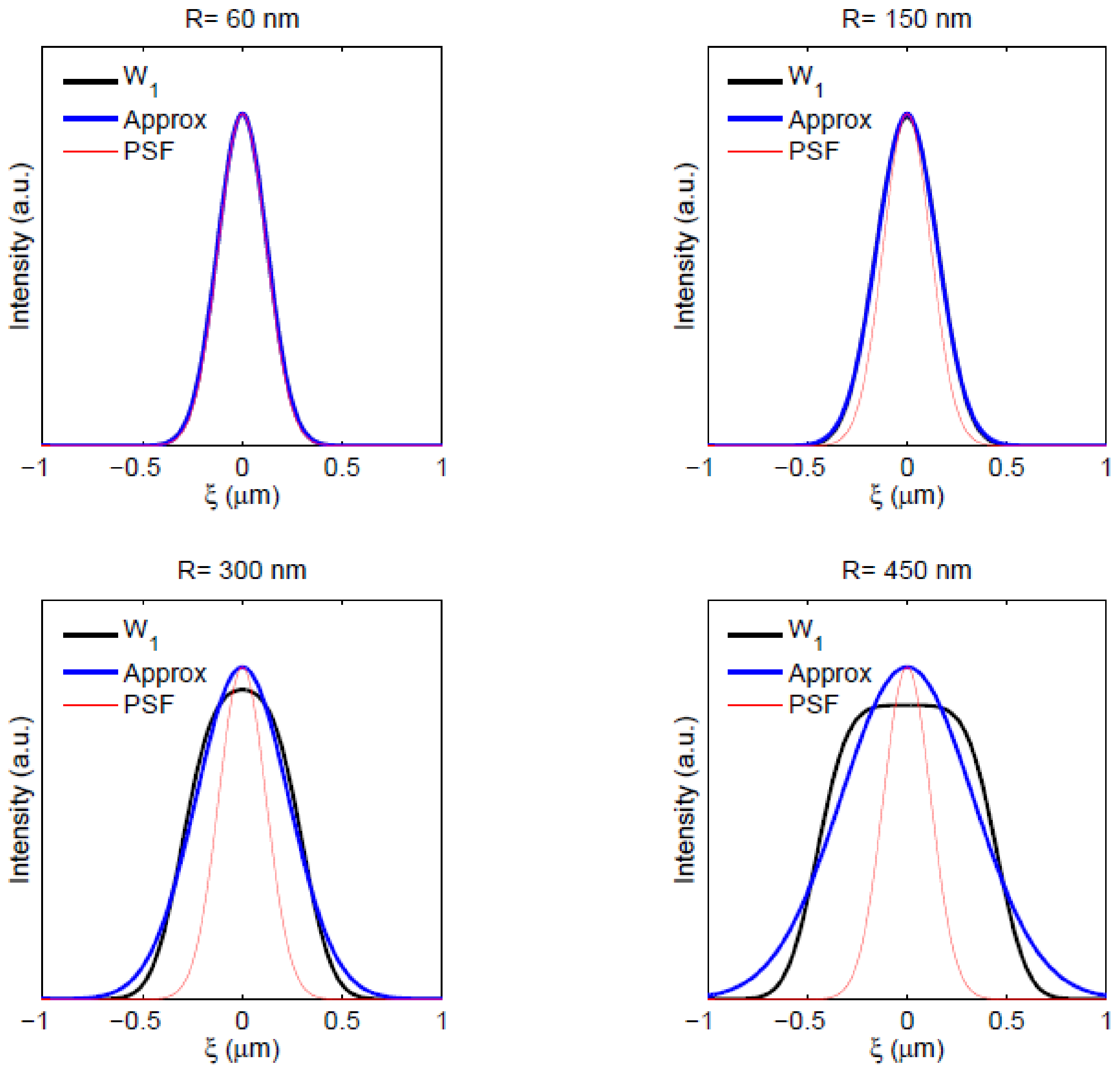

Figure 2 W_1 (black lines) and corresponding Gaussian approximation (blue lines) for different R values. The red lines indicate the PSF (i.e. W).

Where the integral arises from the convolution of the involved functions and its analytic solution involves the error function. Eq. 7 can be approximated by the Gaussian function:

$$W_1(\xi, 0) \propto exp\left\{-\frac{2\xi^2}{w_1^2}\right\} \tag{8}$$

In this regard, Fig. 2 shows the analytic solution of W_1 (black lines) and the corresponding Gaussian fitting curve (blue line) for different R values. Under this approximation, the waist $w_1$is

$$w_1^2 = w^2 + 2R^2 \tag{9}$$

Finally, by combining Eq. 1, 8, 9 and evaluating g at zero time-lag, follows that the intercept of the *i*MSD curve reads $\sigma^2(0) = w_1^2$, thus the particle radius can be estimated as:

$$R^2 = \frac{\sigma^2(0) - w^2}{2} \tag{10}$$

Supporting references

1. Di Rienzo C, Gratton E, Beltram F, & Cardarelli F (2016) Super-Resolution in a Standard Microscope: From Fast Fluorescence Imaging to Molecular Diffusion Laws in Live Cells. In: Super-Resolution Imaging in Biomedicine. *Taylor & Francis Group*:19-47.

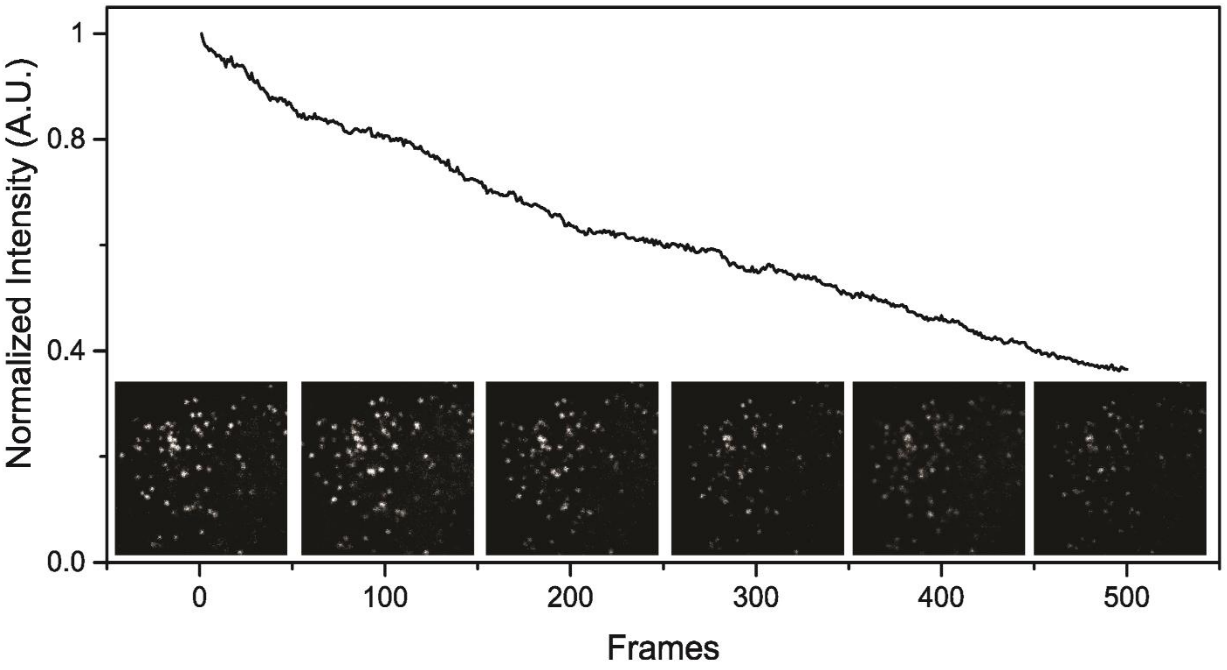

Suppl. Fig. 1. Normalized intensity fluorescence counts of a typical acquisition of transfected ISGs versus frames progression/time, with exemplary images extracted from the acquired movie, at frame n=0, 100, 200, 300, 400, 500.

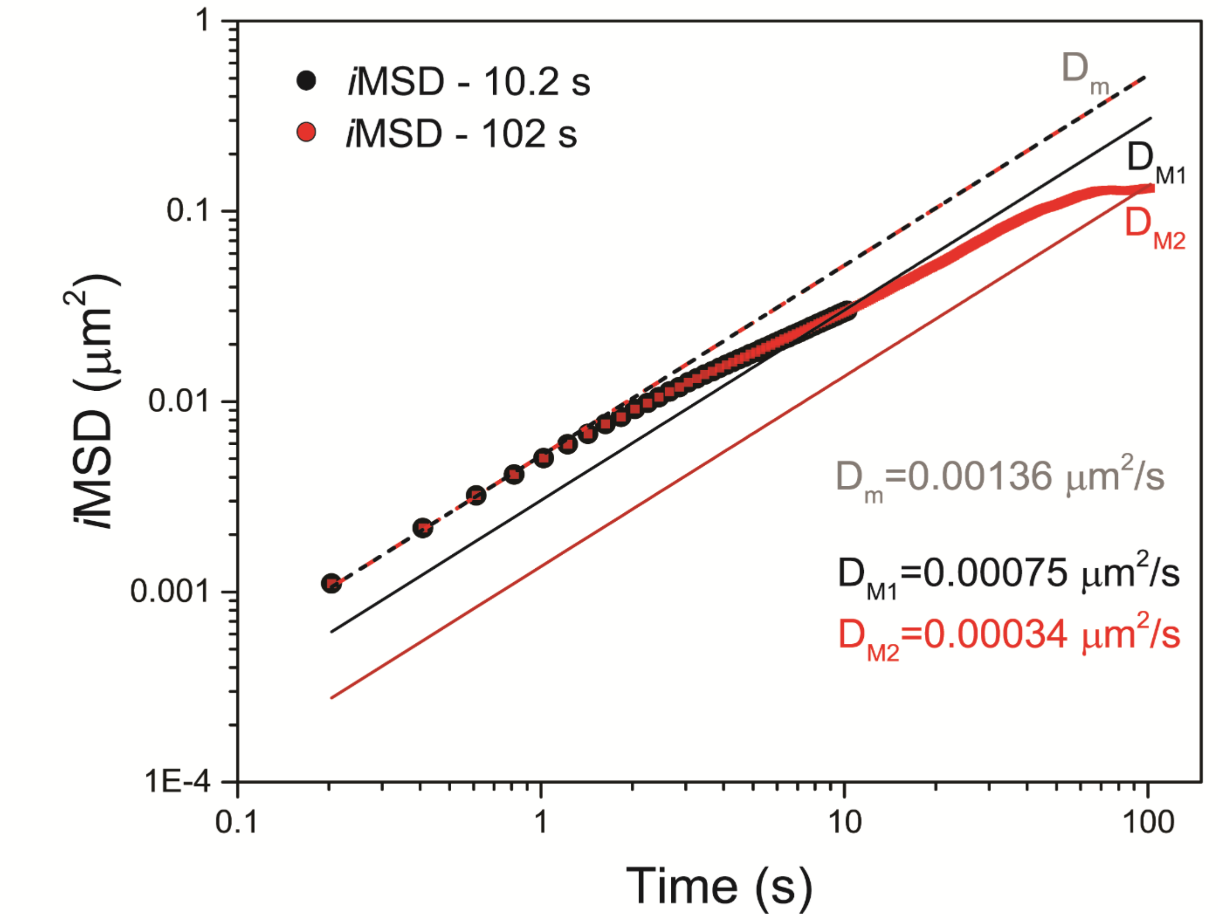

Suppl. Fig. 2. Two iMSD curves of different duration (10.2 s, black dot and 102 s, red dot) are represented in log-log scale. Both curves are characterized by the same value of D_m (short range diffusivity) coefficient, represented by tangent red and black dashed line, while they differ for D_M (long range diffusivity) coefficients, represented by tangent black solid line for shorter curve and tangent red line for the longer one.

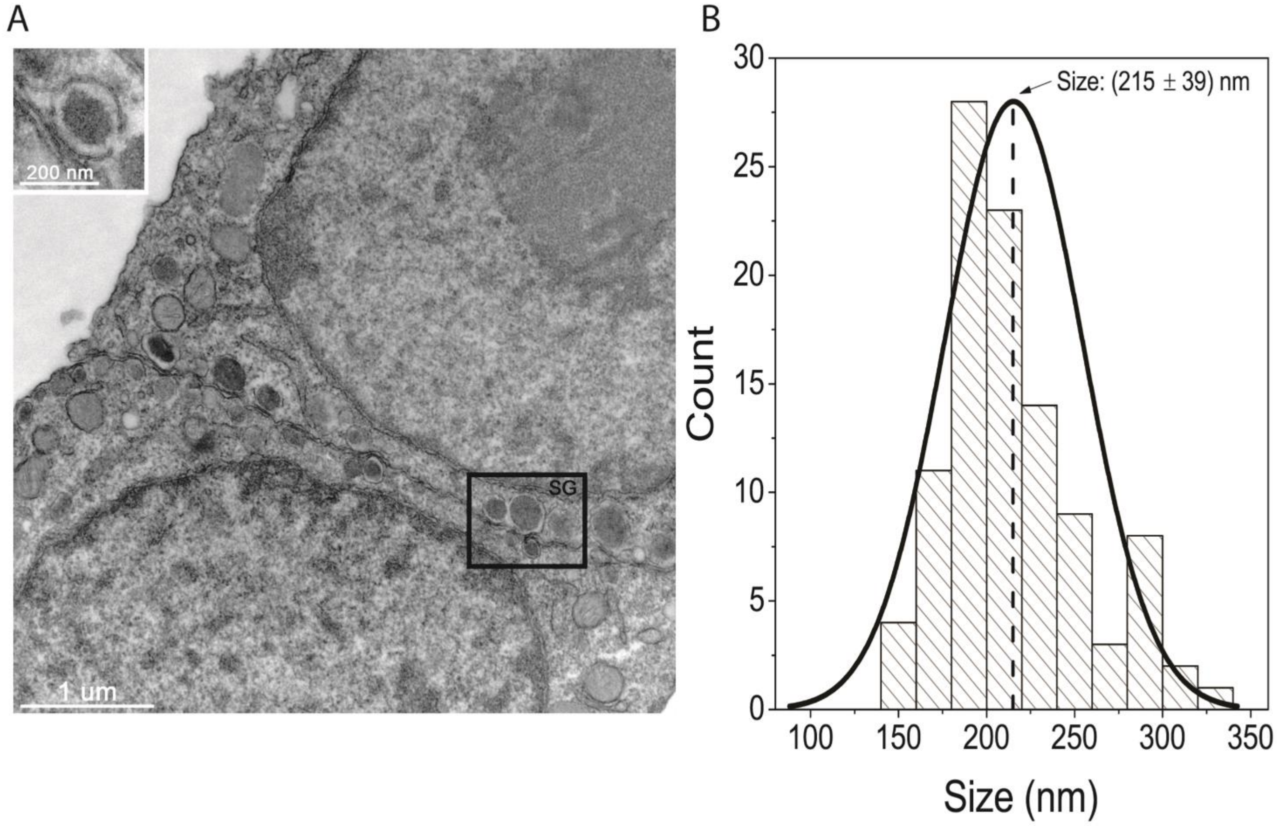

Suppl. Fig. 3. A) Micrograph of INS-1 E cells. Granules (SG) are dense core structure with a well-defined outer membrane (inset). B) Diameter (size) distribution of n=104 analyzed granules. Mean value and standard deviation of the distribution are reported.

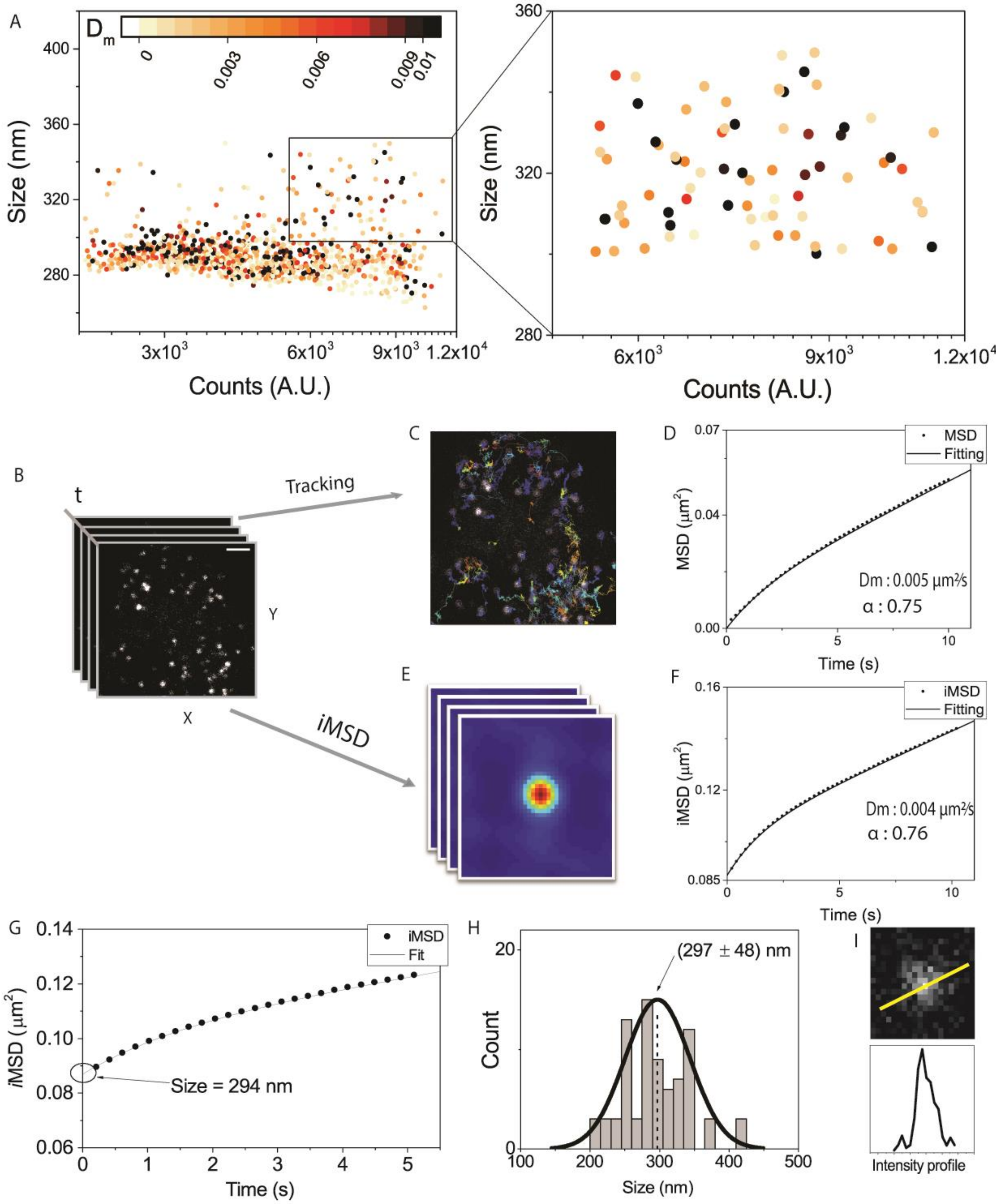

Suppl. Fig. 4. A) Each point in the scatter plot represent a specific localized ISGs for which fluorescence intensity, size, and D_m were estimated by trajectories analysis (n=4 acquisitions). Darkness of the color represents the increasing value of D_m coefficients calculated by tracking analysis. On the right, zoomed region of the scatter plot populated by bigger and more intense ISGs. B) Example of typical acquisition of transfected ISGs (scale bar: 5 μm). C) Representation of trajectories of each granule superimposed to the first frame of the movie, extracted by TrackMate analysis on ImageJ. D) MSD curve obtained by analyzed tracks. Fitting with same equations describe in Methods lead to reported values for D_m and αcoefficients. E) Stack of images representing the evolution of the spatio-temporal correlation function (STICS), obtained by iMSD

custom made Matlab algorhythm (as described in Methods). F) $_{i}$MSD curves obtained for movie in A). Fitting procedures lead to reported values, in agreement to tracking-based retrieved ones. G) Zoomed region of experimental $_{i}$MSD with relative fitting curve. The square root of the y-axis intercept retrieved by fitting is used to estimate the average size (diameter) of imaged ISGs. H) size (diameter) distribution of imaged ISGs. Values were calculated from the first frame of the movie for which $_{i}$MSD curve in (G) was reported. I) Example of size calculation for imaged ISGs. The intensity profile along the yellow line was fitted with a Gaussian function to estimate of spot size.

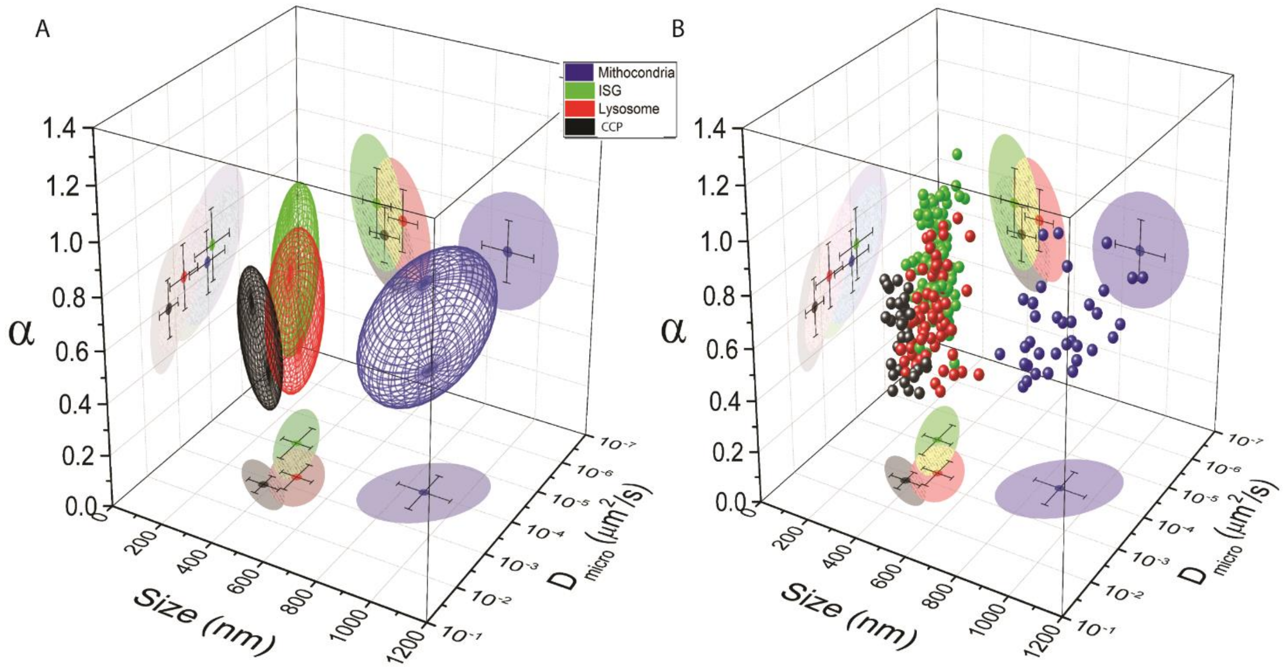

Suppl. Fig. 5. Structural and dynamic fingerprints of subcellular organelles in INS-1 E cells. 3D dynamic fingerprints of Mitochondria (blue), Lysosomes (red) Clathrin coated pits (CCPs, black) and ISGs (green) imaged in INS1 E cells, represented in A) as data points and in B) as 68% confidential ellipsoids. On the walls of the scatter plot the projections of confidence ellipsoids are depicted.

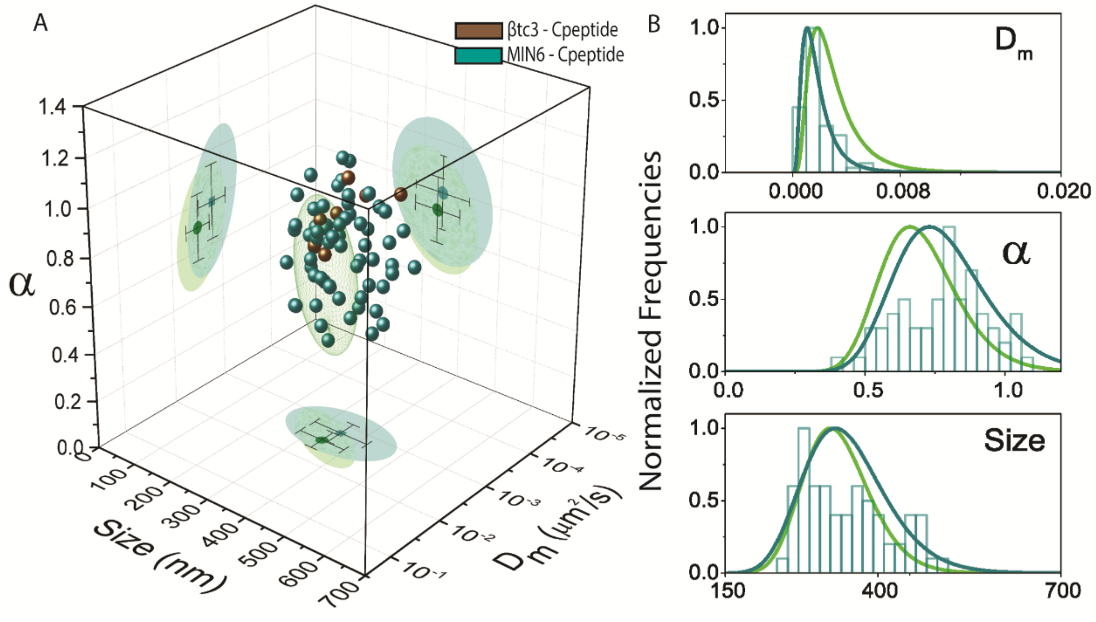

Suppl. Fig. 6. A) Dynamic fingerprint of proinsulin-EGFP ISGs in MIN6 cells (dark cyan) and of proinsulin-EGFP ISGs in βtc3 cells (brown), with reference proinsulin-EGFP ISGs in INS-1 E cells represented as 68% confidence ellipsoid (green). B) Normalized relative distributions of α, D_m and size for proinsulin-EGFP ISGs in MIN6 cells (dark cyan bars and distributions).

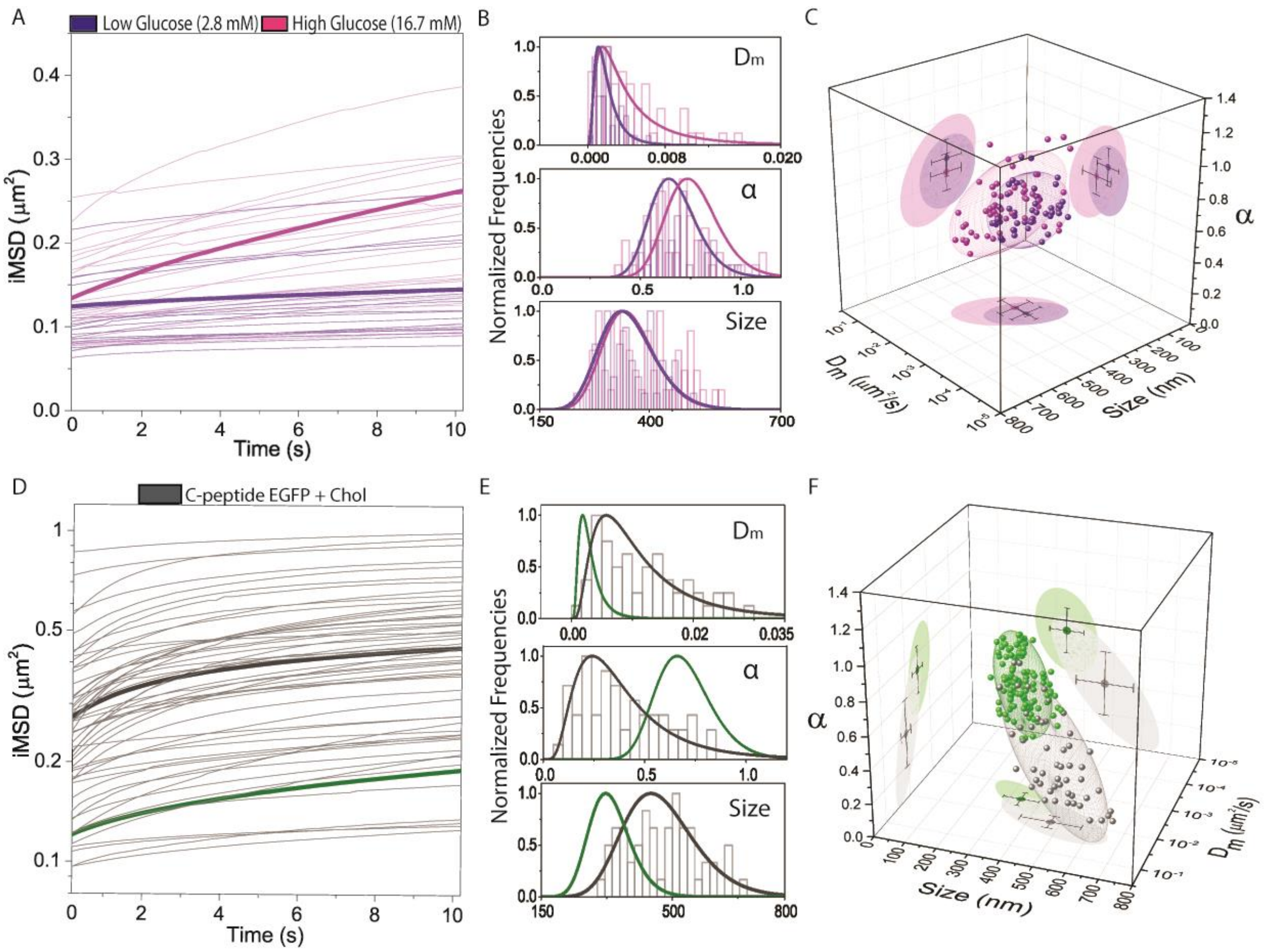

Suppl. Fig. 7. Effect of glucose stimulation and cholesterol overload on ISG fingerprint. A) *i*MSD curves of n=58 proinsulin-EGFP ISGs cultured in low glucose (2.8 mM) medium (average curve in violet),in magenta n=54 *i*MSD curves of proinsulin-EGFP ISGs cultured in high glucose (16.7 mM) medium. B) Normalized relative distributions of D_m α and size for low glucose (violet bars and fit) condition compared to high glucose condition (magenta bars and fit). C) Dynamic fingerprint of proinsulin-EGFP ISGs in low glucose medium compared to proinsulin-EGFP ISGs in high glucose medium. D) *i*MSD curves of n=71 acquisitions of INS-1 E cells transfected with proinsulin-EGFP and treated with MβCD-Chol, with the average curve highlighted in bold grey. The bold green curve represents the reference of untreated cells (same as in Figure 2). E) Normalized relative distributions of D_m α and size for cholestrol-treated cells (grey bars and fit) compared to untreated cells (green reference fit, same as in Figure 2). F) Dynamic fingerprint of cholestrol-treated cells (grey dots) compared to untreated cells (represented as a 68% confidential ellipsoid in green).

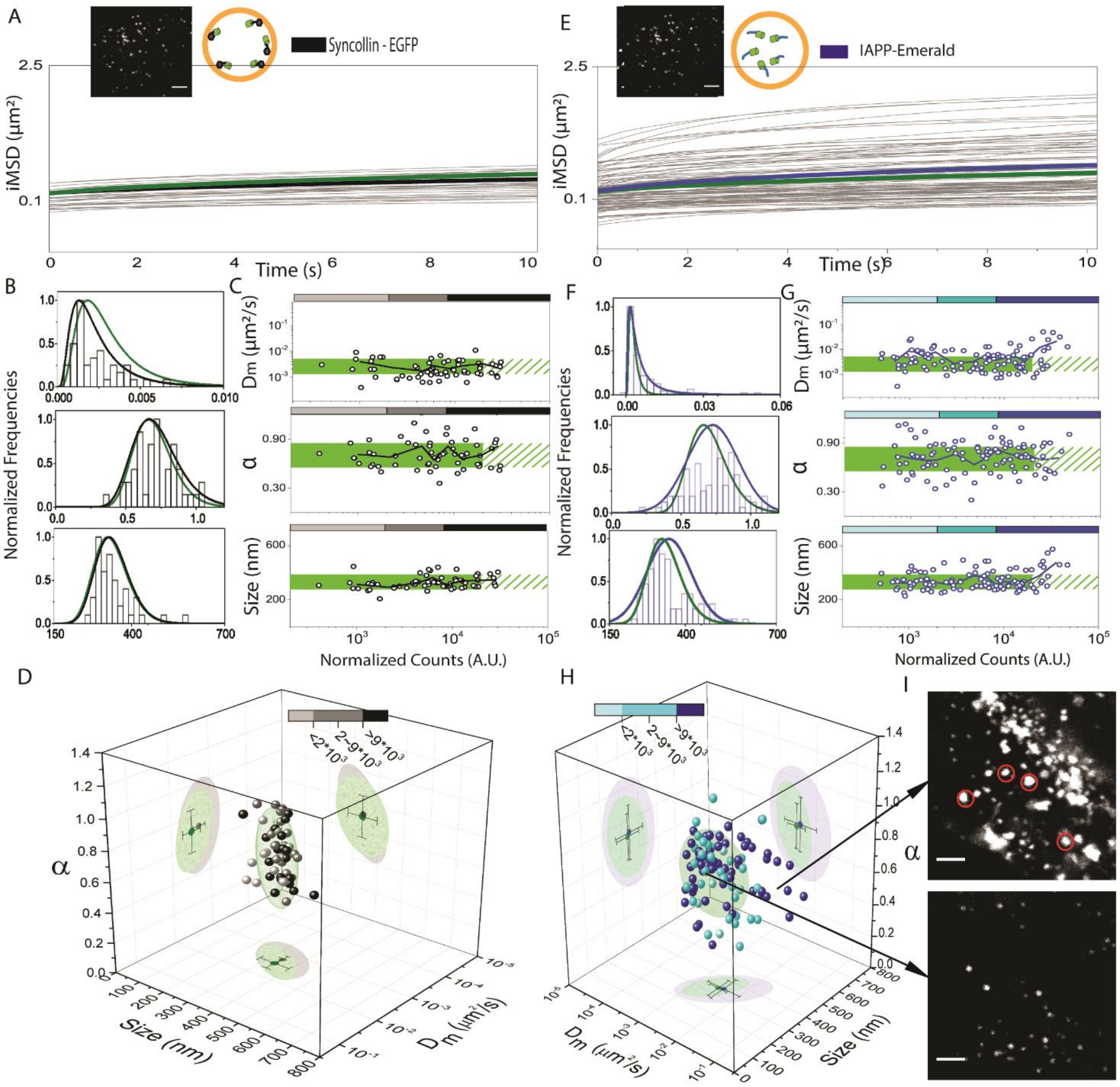

Suppl. Fig. 8. Fingerprints of syncollin-EGFP and IAPP-EGFP labelled ISGs. A) On top, image representing typical acquisition of syncollin-EGFP ISGs cells (scale bar: 2 µm); schematized representation of intraluminal localization of fluorescent protein in syncollin-EGFP transfected ISG and *i*MSD curves of n=48 acquired cells. In black average *i*MSD curve compared to average *i*MSD curve of proinsulin-EGFP ISGs (green). B) Normalized relative distributions of D_m α and Size for syncollin ISGs. C) Fluorescence expression level analysis of syncollin ISGs (black points), as described in Fig 1F. Green area in each plot is defined by average values plus and minus relative SDs of sroinsulin-EGFP ISGs data. C) Dynamic fingerprint of syncollin-EGFP ISGs compared to proinsulin- EGFP ISGs dynamic fingerprint (represented as a 68% confidential ellipsoid in green). Color mapping is used to define three fluorescence expression regimes in which syncollin ISGs points are categorized. E) On top, exemplary portion of cytoplasm of a IAPP-Emerald expressing cell (scale bar:2 µm) and schematized representation of intraluminal fluorescent protein localization in IAPP-Emerald transfected ISG. *i*MSD curves of n=136 acquisitions, with the average highlighted in bold blue (and proinsulin- EGFP average trace in bold green). F) Normalized relative distributions of D_m α and size for IAPP-Emerald expressing cells. G) Fluorescence expression level analysis, of IAPP-Emerald ISGs (blue points), as described in Fig 1F. Green area in each plot is defined by average values plus and minus relative SDs of proinsulin- EGFP ISGs data. H) Structural/dynamic fingerprint of IAPP-labelled ISGs as compared to Proinsulin- EGFP reference. Points are color-coded as a function of the protein expression level. I)Examples of ISGs morphology alteration induced by high expression level of IAPP-Emerald, with exemplary ISGs highlighted in red or green of two distinct observable populations. Scale bar: 2 µm.

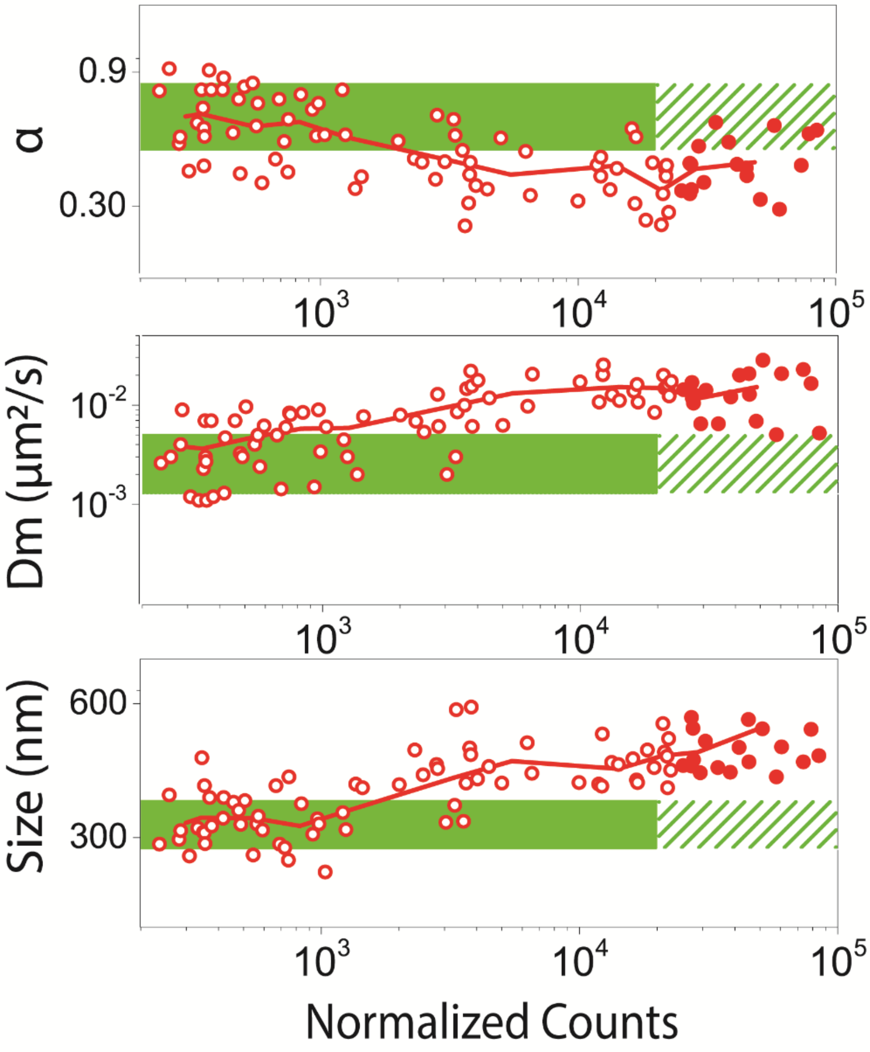

Suppl. Fig. 9. Fluorescence expression level analysis of phogrin-EGFP, as described in Fig 1F. The solid green area in each plot indicates the range of values (mean ±SD) observed in cells transiently transfected with proinsulin-EGFP (and reported in Fig 2C) for experimental expression level, while dashed green areas represent a virtual extension of expression level not covered by experimental data points of proinsulin-EGFP ISGs. Solid red lines represent a moving average of 8 experimental data points; dashed green lines represent standard deviation of all experimental data points. Solid red points represent experimental data points for phogrin-EGFP ISGs exceeding the range of proinsulin-EGFP expression level.

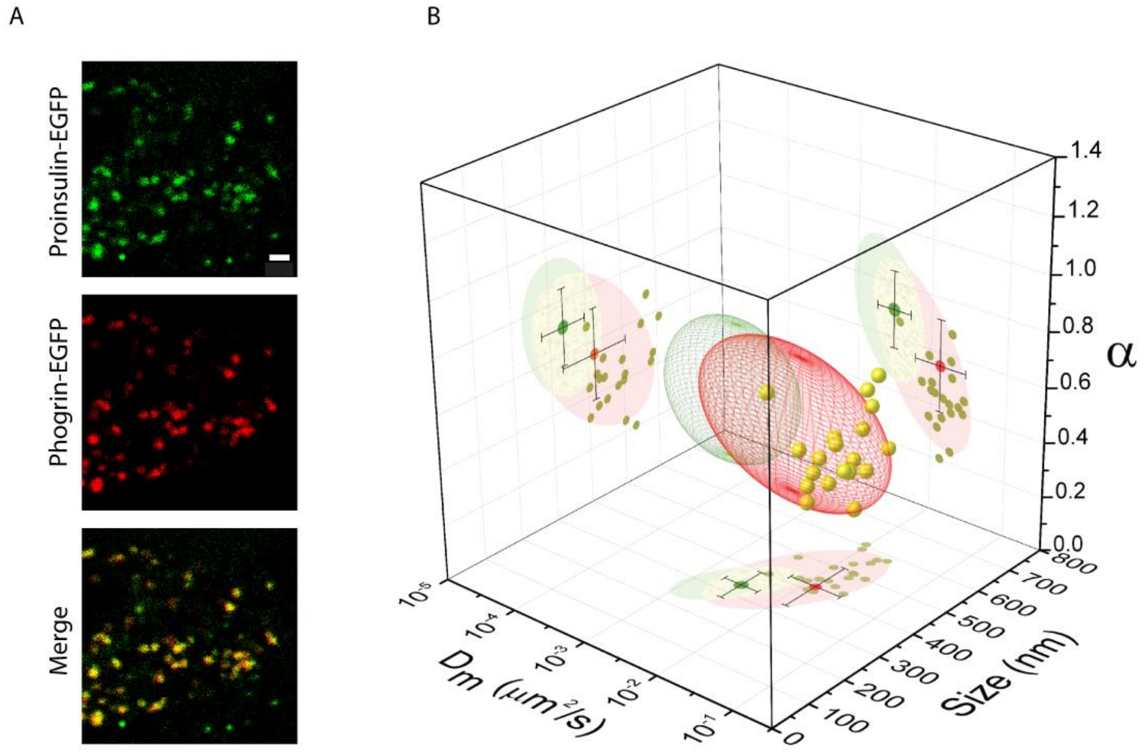

Suppl. Fig. 10. A) Co-transfection of phogrin-mCherry and proinsulin- EGFP (Scale bar: 2µm). B) Dynamic fingerprint of proinsulin- EGFP and phogrin-mCherry co-transfected ISGs (in yellow), compared to proinsulin- EGFP ISGs (68% confidential ellipsoid in green), and phogrin-EGFP ISGs (68% confidential ellipsoid in red).

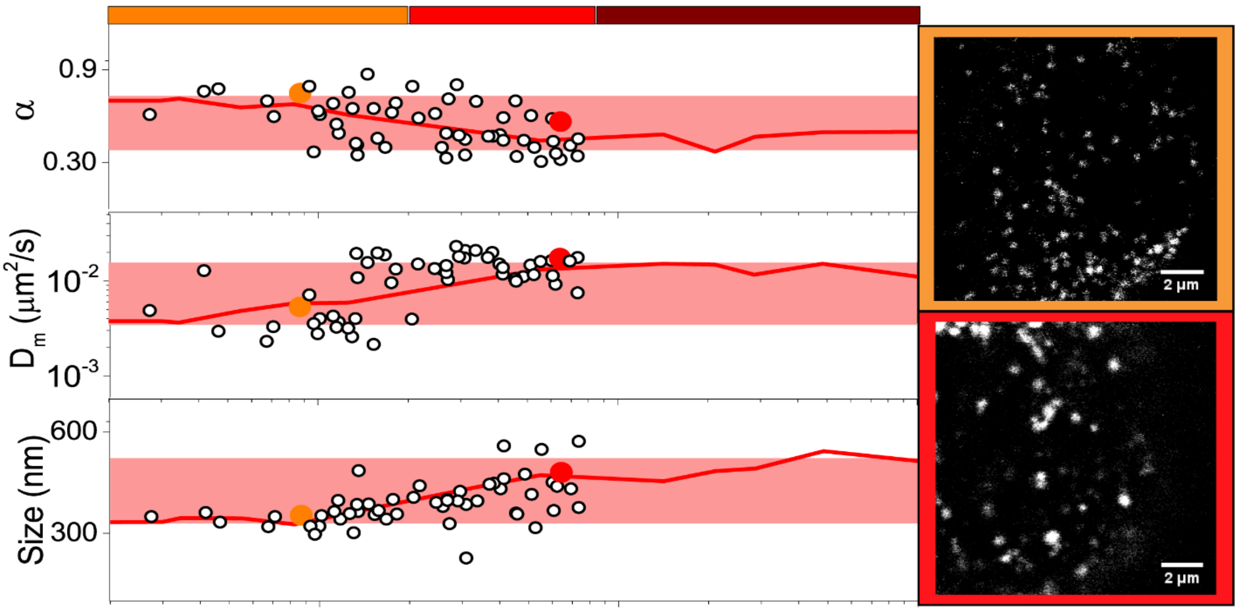

Suppl. Fig. 11. Fluorescence expression level analysis of stably expressed phogrin-EGFP (black points), as described in Fig 1F. The red area in each plot indicates the range of values (mean ±SD) observed in cells transiently transfected with phogrin-EGFP (and reported in Fig. 3C). For two exemplary expression levels (defined in Fig. 3C) two representative cells are highlighted in the plot (orange and red circles) and showed by confocal images on the right.

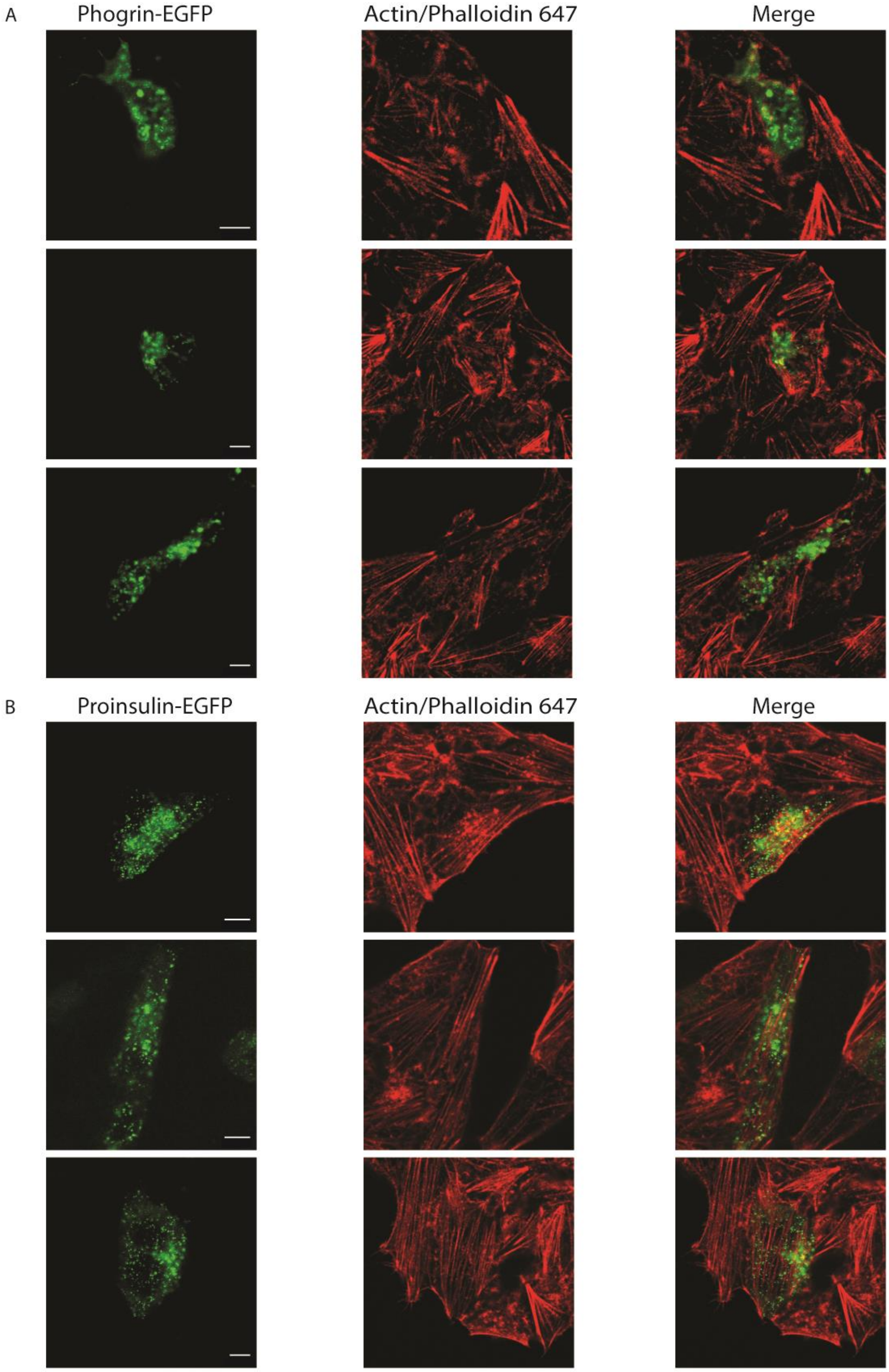

Suppl. Fig. 12 A) Phogrin-EGFP transfected ISGs (green), actin labeled with Phalloidin-647 (red) and overlay of the two channels. B)Proinsulin-EGFP transfected ISGs (green), actin labeled with Phalloidin-647 (red) and overlay of the two channels. Scale bar: 5 µm. Scale bar: 5 µm.

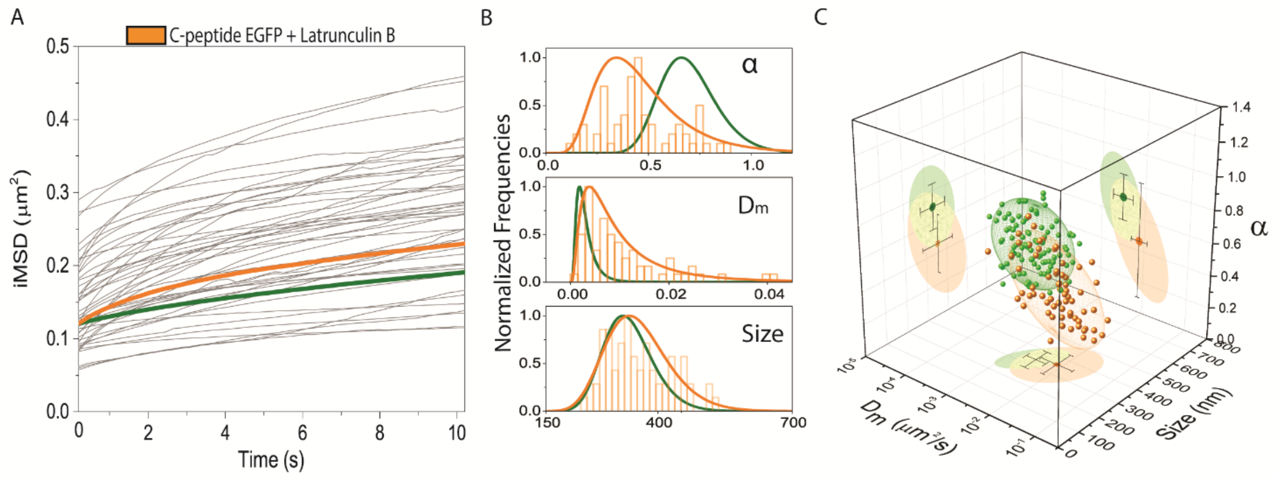

Suppl. Fig. 13 A) *i*MSD curves of n=65 Latrunculin-B treated proinsulin-EGFP ISGs acquisitions. Average *i*MSD curve in orange, in green *i*MSD curve of untreated proinsulin-EGFP ISGs. B) Normalized relative distributions of α, D_m and Size of Latrunculin B treated ISGs (orange) and untreated (green). C) Dynamic fingerprint of Latrunculin B treated proinsulin-EGFP ISGs compared to proinsulin-EGFP ISGs dynamic fingerprint (represented as a 68% confidential ellipsoid in green).

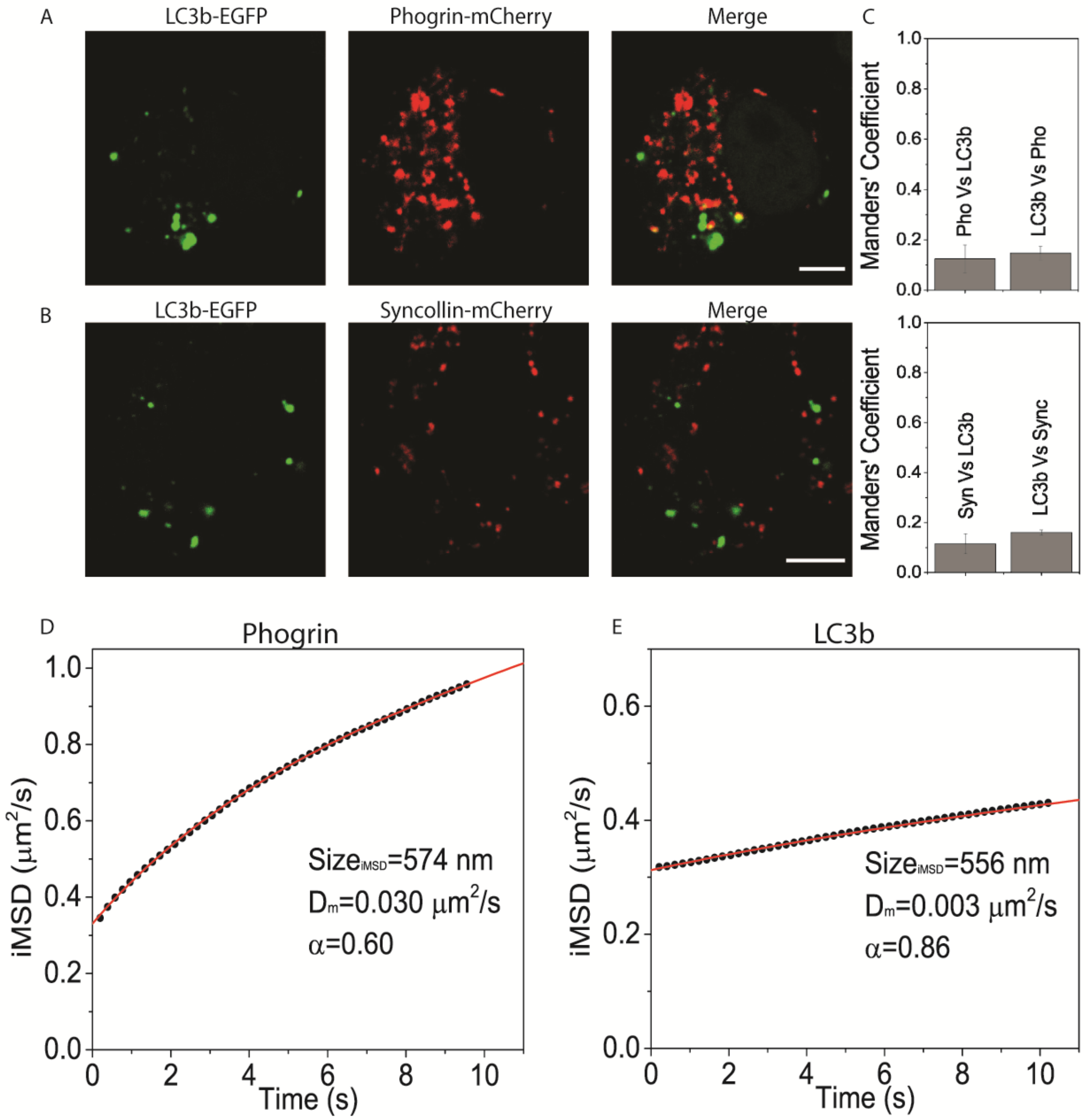

Suppl. Fig 14. A) LC3b-EGFP transfected INS-1 E for autophagosome labelling (green). In red signal of phogrin-mCherry labelled ISGs and merge of the two channel. Scale bar: 2 μm. B) LC3b-EGFP transfected INS-1 E for autophagosome labelling (green). In red signal of syncollin-mCherry labelled ISGs and merge of the two channel. C) Manders' coefficients calculated for two channels acquisition of Phogrin-mCherry and LC3b-EGFP and for Syncollin-mCherry and LC3b-EGFP (n=10 cells for each comparison). D) *i*MSD curve of phogrin-mCherry ISGs channel with fitted values of Size, D_m and α reported. E) *i*MSD curve of LC3b-EGFP channel with fitted values of Size, D_m and α reported.
